# Systematic review and meta-analysis of nutrient supplements for treating sarcopenia in people with chronic obstructive pulmonary disease

**DOI:** 10.1007/s40520-024-02722-w

**Published:** 2024-03-14

**Authors:** Wen-Jian Huang, Chih-Yuan Ko

**Affiliations:** 1 Department of Clinical Nutrition, the Second Affiliated Hospital of Fujian Medical University, No. 34, Zhongshanbei Rd, Licheng District, Quanzhou, 362000 Fujian China; 2 Huidong Center for Chronic Disease Control, Huizhou, 516300 Guangdong China; 3 School of Public Health, Fujian Medical University, Fuzhou, Fujian China

**Keywords:** Chronic obstructive pulmonary disease (COPD), Meta-analysis, Muscle, Nutrient supplements (NS), Sarcopenia

## Abstract

Individuals with chronic obstructive pulmonary disease (COPD) are prone to malnutrition and sarcopenia as a result of nutritional deficiencies and increased energy metabolism. However, the effects of nutrient supplements (NS) on treating sarcopenia in patients with COPD are not well established from systematic evidence. This meta-analysis examined the effect of NS on sarcopenia in patients with COPD. A systematic search of multiple databases was conducted, and 29 randomized controlled trials involving 1625 participants (age, mean [SD] = 67.9 [7.8] years) were analyzed. NS demonstrated significant improvements in body weight (MD,1.33 kg; 95% CI, 0.60, 2.05 kg; P = 0.0003; I^2^ = 87%), fat-free mass index (MD, 0.74 kg/m^2^; 95% CI, 0.21, 1.27 kg/m^2^; P = 0.007; I^2^ = 75%), and 6-min walk test (MD, 19.43 m; 95% CI, 4.91, 33.94 m; P = 0.009; I^2^ = 81%) compared with control. However, NS had nonsignificant effects on handgrip strength (SMD, 0.36; 95% CI, − 0.15, 0.88; P = 0.16; I^2^ = 87%) and quadriceps muscle strength (SMD, 0.11; 95% CI, −  0.06, 0.27; P = 0.20; I^2^ = 25%) compared with the control. In conclusion, NS may be an effective treatment for improving body composition and physical performance in COPD. Future studies should explore the effects of intervention durations, specific NS types, or combined training in patients with COPD and sarcopenia.

**Supplementary Information:**

The online version contains supplementary material available at 10.1007/s40520-024-02722-w.

## Introduction

Chronic obstructive pulmonary disease (COPD) is caused by repeated exposure to noxious stimuli, such as tobacco smoke and air pollutants [1]. COPD is a major cause of morbidity, mortality, and healthcare use worldwide, particularly as life expectancy increases and ambient air pollution worsens because of rapid economic development [2]. According to the Global Burden of Diseases Study, approximately 3.3 million deaths and 74.4 million disability-adjusted life years were attributed to COPD in 2019 [3].

Muscle atrophy (i.e., secondary sarcopenia) is a result of physiological changes specific to COPD [4]. Approximately 27.5% of patients with COPD suffer from sarcopenia, particularly those at Global Initiative for COPD (GOLD) stages III–IV [5]. Patients with COPD and sarcopenia may have a worse prognosis because of impaired respiratory and peripheral muscle function, aggravating dyspnea, and reduced exercise capacity [6].

With several effective treatments for COPD, effective management strategies can alleviate the disease burden and improve patients’ quality of life [3, 7]. Currently, nutritional strategies are one of the cornerstones for managing COPD-related sarcopenia to reduce the effects of COPD on muscles and regulate the metabolism of muscle protein in patients with COPD [8, 9].

In a series of meta-analyses, nutritional supplements, with or without exercise, reduced the risk of sarcopenia in adults [10–12]. Nutritional support for muscle mass or muscle function in patients with COPD has been systematically reviewed; however, the pooled analysis of outcome measurements in sarcopenia is incomplete [13, 14]. A review showed improved fat-free mass (FFM), muscle strength, and physical performance in patients with COPD; however, the findings were narratively recapped [15].

Nutrient supplements (NS) refer to a type of health products containing one or several natural plant or animal nutrients or synthetic nutrients [16]. NS are used to compensate for nutrient deficiencies in people with COPD; however, their effects remain unclear. In addition, patients’ intervention duration, rehabilitation duration, or NS type may have affected their outcomes. However, evidence on the quantitative effects of NS interventions is limited. Thus, this systematic review and meta-analysis examined the effects of NS on the treatment of sarcopenia in patients with COPD.

## Methods

### Search strategy

We performed the review according to the Preferred Reporting Items for Systematic Reviews and Meta-Analyses (PRISMA) 2020 statement and registered it in PROSPERO International prospective register of systematic reviews (ID: CRD42022337646) [17].

The following electronic databases were searched from inception until July 5, 2022: PubMed, Embase, the Cochrane Library, Web of Science, Ovid, Scopus, and the International Clinical Trials Registry Platform. Publication dates and geographical area were not restricted. Keywords in accordance with PICOS structure, as follows: population: COPD patients; intervention: NS; comparison: no NS; outcome: sarcopenia; study design: randomized controlled trials (RCTs). The detailed search strategy was showed in Table S1.

### Eligibility criteria

Articles included in the study were: (i) original studies written in English; (ii) designed as RCTs; (iii) examining subjects with COPD; (iv) using NS as an intervention (including those concurrently using or comparing it with standard diet and/or dietary counseling); and (v) reporting measurements of at least one of the following: muscle mass, muscle strength or physical performance.

We excluded articles when they failed to meet the inclusion criteria or met one of the following exclusion criteria: (i) the intervention was enteral tube feeding, parenteral nutrition, dietary counseling alone, or snacks; (ii) the nutritional intervention was combined with a pharmaceutical intervention (e.g., with anabolic steroids); (iii) the average duration of the intervention was less than two weeks; (iv) the study design was an animal trial, observational trial, case report, opinion letter, literature review, systematic review, or meta-analysis; (v) the study had a high risk of overall bias; and (vi) data extraction was not possible.

### Article selection

All articles were imported into EndNote software, and duplicates were removed. Two researchers independently screened titles and abstracts and read all potentially eligible publications. Any uncertainties in the selection process were discussed and settled with a third investigator.

### Data extraction and quality assessment

For each study, a researcher extracted data using a pre-determined data form that included the first author’s last name, publication data, country, participant characteristics (age, gender, and health status), intervention type and dose, outcome measurements, and other baseline information. Data extraction was independently verified by other authors.

To assess the differences between the experimental and control groups, changes in mean and standard deviation (SD) were employed as summary statistics. GetData Graph Digitizer version 2.24 was utilized for extracting values from graphs when numerical data were not directly available. For studies that did not report changes in SD, attempts were made to contact the corresponding authors for additional information. Following the guidelines of the Cochrane Handbook for Systematic Reviews of Interventions, in cases where these efforts were unsuccessful, correlation coefficients (corr) were calculated to estimate the changes in SD [18]:

$${\text{Corr = (SD}}_{{{\text{baseline}}}}^{{2}} {\text{ + SD}}_{{{\text{final}}}}^{{2}} - {\text{SD}}_{{{\text{change}}}}^{{2}} {)/(2} \times {\text{SD}}_{{{\text{baseline}}}} \times {\text{SD}}_{{{\text{final}}}} {)}$$

The following formula was then applied to calculate the changes in SD:

$${\text{SDchange = }}\sqrt {\left( {{\text{SD}}_{{{\text{baseline}}}}^{{2}} {\text{ + SD}}_{{{\text{final}}}}^{{2}} - {2} \times {\text{Corr}}\;{\text{SD}}_{{{\text{baseline}}}} \times {\text{SD}}_{{{\text{final}}}} } \right)}$$

Using version 2 of the Cochrane risk-of-bias tool (RoB 2) for RCTs, two researchers independently assessed the quality of included studies. The RoB 2 covers five areas of bias: randomization process, deviations from the intended interventions, missing outcome data, measurement of the outcome, and selection of the reported result. A risk-of-bias judgment were made for each domain and overall as low, some concerns, or high [19].

### Data synthesis and analysis

An analysis of pre- and post-treatment changes for intervention and control groups were performed to examine the efficacy of NS on sarcopenia, generating pooled values in form of mean differences (MDs) and 95% confidence intervals (CIs). When the units of outcome measurements were inconsistent, standardized mean differences (SMDs) were used. Based on I^2^ statistics, the heterogeneity of outcomes between studies was determined. I^2^ ≤ 50% was considered low heterogeneity, and the data were pooled using a fixed-effect model; while I^2^ > 50%, the data were pooled using a random-effect model. The results of this meta-analysis were visualized using forest plots.

Moreover, we conducted multiple sensitivity analyses by removing one study at a time to determine the effect of each included study on the pooled effect. We conducted subgroup analyses based on intervention periods, pulmonary rehabilitation presence or absence, and NS types. Review Manager (RevMan 5.3) was used for all statistical analyses.

## Results

### Search and study characteristics

**Fig. 1:**
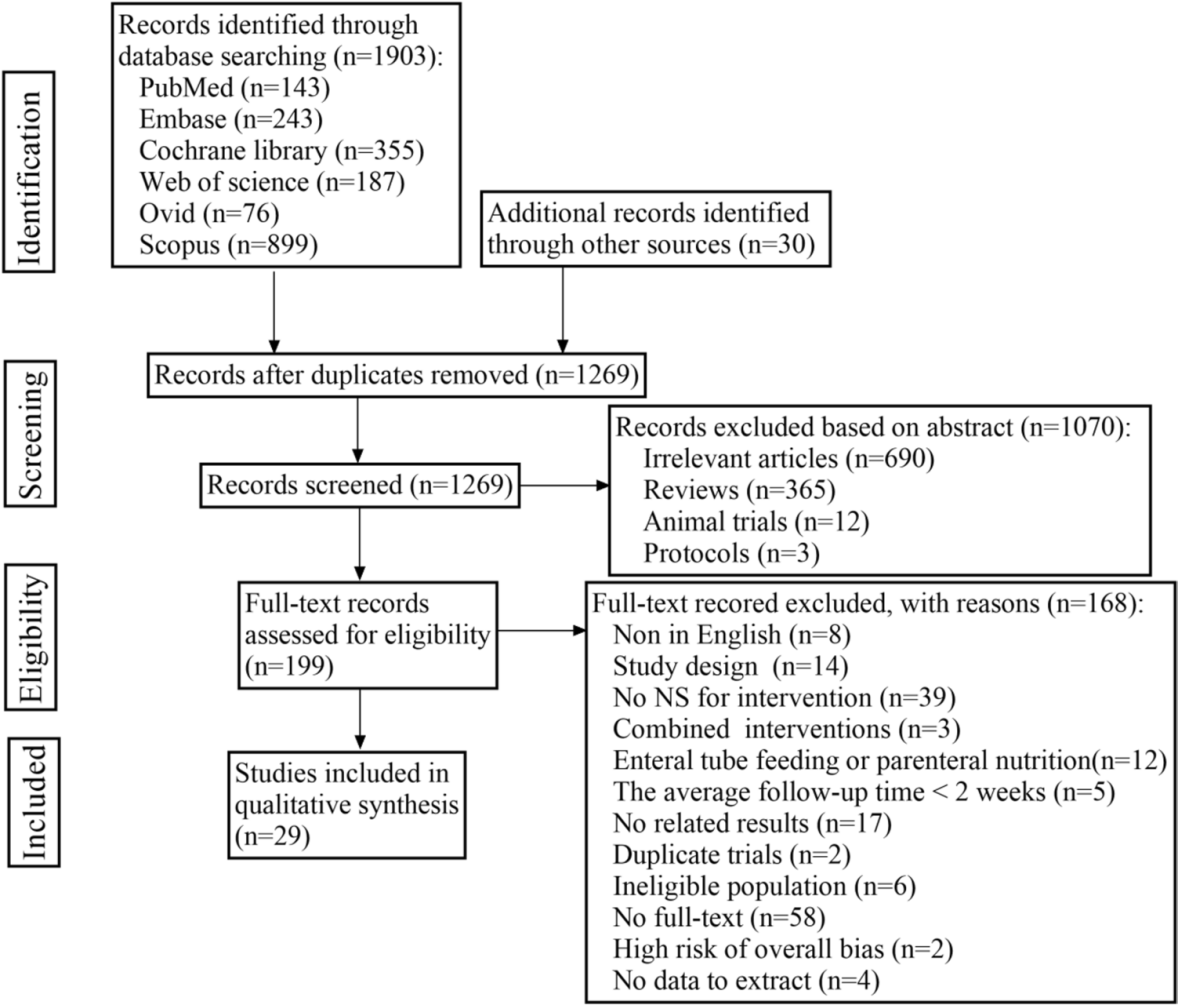
Flow diagram of the search strategies for the study selection

The selection process of studies for the meta-analysis is shown in Fig. 1. In total, 1903 articles were identified through searches, of which 29 were included in the final analysis [9, 20–47]. The overall mean ± SD age of 1625 participants in 29 studies was 67.9 ± 7.8 years. Moreover, 817 participants received NS, and 808 received usual care or placebo.

The meta-analysis includes 29 studies, summarized in Table 1. There were three studies that had a gender-specific design for COPD patients who were male [23, 30, 36]; over 70% of the COPD patients in eight studies were male [20, 25, 31, 34, 37, 39, 41, 47]; two studies did not report the number of men and women [33, 35]. Except for two studies on admission, almost all participants with COPD were stable [21, 41]. Moreover, participants in ten studies were malnourished, manifested as low body mass index (BMI) criteria, low muscle mass, or recent involuntary weight loss [21, 22, 26–28, 34, 37, 38, 41, 47].

**Table 1:** Characteristics of included studies

			Intervention details				Outcomes		
Study (author, year, country, ref)	Sample size (n)	Age (y) Mean ± SD Sex(M/F)	Composition	Amount	Control details	Measured time point	Muscle mass	Strength	Physical performance
Zanforlini et al., 2022, Italy [20]	NS n = 25 CG n = 24	NS 73.0 ± 8.9 CG 72.2 ± 11.0 Sex n = 38/11	Magnesium citrate	300 mg qd (energy: NR)	Maltodextrin (energy: NR)	Baseline Posttest: 24wk	/	HGS; QMS	6-MWT
Deutz et al., 2021, United States [21]	NS n = 109 CG n = 105	NS 74.5 ± 7.3 CG 75.2 ± 7.6 Sex n = 101/113	50EN% CHO, 22EN% protein, 28EN% fat, HMB 1.5 g/ portion and 26 other essential vitamins and minerals	237 ml bid (700 kcal/d)	100EN% CHO, and 10 mg vitamin C, but no other macro- or micronutrients (96 kcal/d)	Baseline Posttest: 4wk	/	HGS	/
de Bisschop et al., 2021, France [9]	NS n = 25 CG n = 29	NS 65.4 ± 8.8 CG 64.4 ± 8.0 Sex n = 36/18	82EN% protein, 7EN% CHO, 5EN% fat, 2.67 g fiber and 0.3 g salt (contained 4.3 g BCAA)	25 g qd (approx. 85 kcal/d)	0.7 g proteins, 2.05 g glucids, 0.38 g lipids and 19 g fibers (approx. 14 kcal/d)	Baseline Posttest: 4wk	BW; BMI	QMS	6-MWT
Aldhahir et al., 2021, UK [22]	NS n = 22 CG n = 22	NS 75 ± 6 CG 70 ± 9 Sex n = 28/16	24EN% protein, 41EN% CHO and 35EN% fat	125 ml bid (600 kcal/d)	100EN% CHO (200 kcal/d)	Baseline Posttest: 6wk	BW; FFM; FFMI	HGS	ISWT; STS5; PAL
Ahmadi et al., 2020, Iran [23]	NS n = 23 CG n = 23	NS 62.08 ± 7.0 CG 63.47 ± 7.24 Sex n = 46/0	275 mg elemental magnesium, 685 mg vitamin C and 15.9 g whey protein	250 ml pd (113.6 kcal/d)	UC	Baseline Posttest: 8wk	BW; BMI; FFM; FFMI; CC; AC	HGS	/
Gouzi et al., 2019, France [24]	NS n = 31 CG n = 26	NS 62.4 ± 6.5 CG 61.1 ± 8.7 Sex n = 28/29	30 mg α-tocopherol, 180 mg ascorbate, 15 mg zinc gluconate and 50 μg selenomethionine	One portion pd (energy: NR)	NR	Baseline Posttest: 4wk	BMI; FFMI	QMS	6-MWT
De Benedetto et al., 2018, Italy [25]	NS n = 45 CG n = 45	NS 73 ± 7 CG 73 ± 7 Sex n = 68/22	170 mg creatine and 160 mg coenzyme QTer®	One portion bid (energy: NR)	NR	Baseline Posttest: 8wk	BMI	/	6-MWT
Calder et al., 2018, Sweden [26]	NS n = 22 CG n = 23	NS 69.2 ± 6.3 CG 69.7 ± 8.2 Sex n = 23/22	10 μg 25-hydroxy-vitamin D3, 10 g whey protein concentrate and minimum 2.0 g n-3 PUFAs	200 ml bid (approx.460 kcal/d)	No 25-hydroxyvitamin D3, milk protein in place of pure whey protein and sunfower oil instead of n-3 PUFAs-enriched fish oil (approx. 400 kcal/d)	Baseline Posttest: 12wk	BW; BMI; CC	/	6-MWT
van de Bool et al., 2017, Netherlands [27]	NS n = 42 CG n = 39	NS 62.8 ± 8.4 CG 62.2 ± 8.1 Sex n = 41/40	20EN% protein, 60EN% CHO, and 20EN% fat, and enriched with leucine, n-3 PUFA, and vitamin D	125 ml bid/tid (375 or 562.5 kcal/d)	A flavoured non‐caloric aqueous solution (approx. 0 kcal/d)	Baseline Posttest: 16wk	BW	QMS	CET; 6-MWT; PAL
Rafiq et al., 2017, Netherlands [28]	NS n = 24 CG n = 26	NS 64 [61–66] CG 61 [58–66] Sex n = 26/24	Vitamin D3	1200 IU pd (energy: NR)	NR	Baseline Posttest: 24wk	BMI	HGS	6-MWT
Paulin et al., 2017, Brazil [29]	NS n = 8 CG n = 8 NS + Ex n = 8 CG + Ex n = 8	NS 63.4 ± 5.2 CG 58.1 ± 10.3 NS + Ex 56.5 ± 5.0 CG + Ex 65.2 ± 6.0 Sex n = 16/16	Vitamin B12	500 mg pd (energy: NR)	Maltodextrin (energy: NR)	Baseline Posttest: 8wk	/	/	CET
Pirabbasi et al., 2016, Malaysia [30]	NS n = 13 CG n = 18	NS 64.5 ± 10.2 CG 64.17 ± 8.3 Sex n = 31/0	Vitamin C	500 mg pd (energy: NR)	UC	Baseline Posttest: 24wk	BMI; FFM; FFMI	/	/
Khan et al., 2016, India [31]	NS n = 30 CG n = 30	NS 55.0 ± 10.4 CG 53.3 ± 10.8 Sex n = 54/6	55EN% CHO, 45EN% protein and 0EN% fat	15 g bid (90 kcal/d)	UC	Baseline Posttest: 12wk	BW; BMI; AC	/	6-MWT
Ahnfeldt-Mollerup et al., 2015, Denmark [32]	NS n = 28 CG n = 25	NS 67 ± 9.7 CG 70 ± 7.3 Sex n = 23/30	Protein bars (27.6EN% protein, 43.3EN% CHO, 28EN% fat, enriched with EAAs and immunonutrition)	35 g bid (269.6 kcal/d)	Isocaloric placebo bar without the protein (269.6 kcal/d)	Baseline Posttest: 9wk	/	QMS	ESWT
Raizada et al., 2014, India [33]	NS n = 15 CG n = 15	NR	50EN% CHO, 34.6EN% fat, 15.4EN% protein, fibre, vitamins, and minerals	114 g/d (500 kcal/d)	UC	Baseline Posttest: 6wk	BW; BMI; AC	/	6-MWT
Gurgun et al., 2013, Turkey [34]	NS n = 15 CG n = 15	NS 64.0 ± 10.8 CG 66.8 ± 9.6 Sex n = 28/2	53.3EN% CHO, 30EN% fat and 16.7EN% proteins	250 ml tid (energy: NR)	UC	Baseline Posttest: 8wk	BW; BMI; FFMI; mid-thigh CSA	/	6-MWT; ISWT; ESWT
Marinari et al., 2013, Italy [35]	NS n = 30 CG n = 25	NS 73.2 ± 8.7 CG 73.9 ± 7.7 Sex NA	170 mg creatine and 160 mg coenzyme Q-Ter	One portion bid (energy: NR)	NR	Baseline Posttest: 8wk	BMI; FFMI	/	6-MWT
Bjerk et al., 2013, USA [36]	NS n = 18 CG n = 18	NS 67.6 ± 7 CG 68 ± 8 Sex 36/0	Vitamin D3	2000 IU pd (energy: NR)	NR	Baseline Posttest: 6wk	/	/	SPPB
Sugawara et al., 2012, Japan [37]	NS n = 17 CG n = 14	NS 77.4 ± 5.2 CG 77.1 ± 5.8 Sex 29/2	20EN% protein, 25EN% fat, 53.2EN% CHO, 1.8EN% food fibers, and BCAA/AAA ratio 3.7	200 ml bid (400 kcal/d)	UC	Baseline Posttest: 12wk	BW; FFM; FFMI	/	6-MWT
Dal Negro et al., 2012, Italy [38]	NS n = 44 CG n = 44	NS 75 ± 5 CG 73 ± 8 Sex 61/27	EAAs	4 g bid (43.8 kcal/d)	Isocaloric placebo (43.8 kcal/d)	Baseline Midtest: 4wk Posttest: 12wk	BW; BMI; FFM; FFMI	HGS	PAL
Hornikx et al., 2012, Belgium [39]	NS n = 25 CG n = 25	NS 67 ± 8 CG 69 ± 6 Sex 38/12	Vitamin D3	Corresponding to 3300 IU pd (energy: NR)	Arachidis oleum (energy: NR)	Baseline Posttest: 12wk	/	QMS	6-MWT
Laviolette et al., 2010, Canada [40]	NS n = 12 CG n = 10	NS 62.9 ± 10.1 CG 67.6 ± 4.4 Sex 14/8	20 g pressurized whey and 20 g CHO	120 ml pd (160 kcal/d)	20 g casein and 20 g CHO (160 kcal/d)	Baseline Midtest: 8wk Posttest: 16wk	BW; mid-thigh CSA	QMS	CET
Baldi et al., 2010, Italy [41]	NS n = 14 CG n = 14	NS 73.1 ± 6.0 CG 70.1 ± 5.8 Sex 20/8	EAAs (high BCAA)	4 g bid (energy: NR)	UC	Baseline Posttest: 12wk	BW; FFM	/	/
Deacon et al., 2008, UK [42]	NS n = 38 CG n = 42	NS 67.6 ± 7.4 CG 68.3 ± 8.2 Sex 50/30	Creatine	3.76 g pd (energy: NR)	Lactose (energy: NR)	Baseline Posttest:7wk	BW; FFM	QMS	ISWT, ESWT
Faager et al., 2006, Sweden [43]	NS n = 13 CG n = 10	NS 64 ± 6 CG 67 ± 6 Sex 10/13	Creatine	0.07 g/kg weight pd (energy: NR)	Glucose (energy: NR)	Baseline Posttest:8wk	BW; BMI	HGS; QMS	ESWT
Broekhuizen et al., 2005, Netherlands [44]	NS n = 51 CG n = 51	NS 64 ± 10 CG 62 ± 8 Sex 71/31	PUFA	9 g/d (81 kcal/d)	Placebo:80% palm oil and 20% sunflower oil (81 kcal/d)	Baseline Posttest:8wk	BW; FFM	QMS	/
Fuld et al., 2005, UK [45]	NS n = 18 CG n = 20	NS 61.7 ± 8 CG 63.7 ± 9.7 Sex 23/15	Creatine	5 g pd (energy: NR)	Glucose polymer (energy: NR)	Baseline Posttest:12wk	BW; FFM	/	ISWT; ESWT
Steiner et al., 2003, UK [46]	NS n = 42 CG n = 43	NS 66 ± 9.0 CG 68 ± 8.0 Sex 32/53	60EN% CHO, 20EN% fat, 20EN% protein	125 ml tid (570 kcal/d)	Flavoured non-nutritive placebo (0 kcal/d)	Baseline Posttest:7wk	BW; BMI; FFM	HGS; QMS	ISWT; ESWT
Lewis et al., 1987, USA [47]	NS n = 10 CG n = 11	NS 65.1 ± 9.2 CG 59.3 ± 9.3 Sex 15/6	40EN% CHO, 45EN% fat, 15EN% protein	240–480 ml (480–960 kcal/d)	UC	Baseline Posttest: 8wk	BW	HGS	/

*NS* nutrient supplements, *CG* control group, *NR* not reported, *HGS* handgrip strength, *QMS* quadriceps muscle strength, *6-MWT* 6-min walk test, *EN%* energy percent, *CHO* carbohydrates, *HMB* beta-hydroxy-beta-methylbutyrate, *BCAA* branched chain amino acid, *BW* body weight, *BMI* body mass index, *FFM* fat-free mass, *FFMI* fat-free mass index, *ISWT* incremental shuttle walk test, *STS5* five-repetition sit-to-stand test, *PAL* physical activity level, *UC* usual care, *CC* calf circumference, *AC* arm circumference, n-3 *PUFAs* n-3 polyunsaturated fatty acids, *CET* cycle endurance time, *Ex* exercise, *EAAs* essential amino acids, *ESWT* endurance shuttle walk test, mid-thigh *CSA* mid-thigh cross-sectional area, *SPPB* short physical performance battery, *AAA* aromatic amino acid

The intervention periods ranged from 4 to 24 weeks. NS interventions were compared with usual care or placebo supplements in all studies. Nutrition supplements were diverse among included studies. Six studies were focused on providing energy-type NS, with prescribed energy ranging from 90 to 960 kcal/d [22, 31, 33, 34, 46, 47]. Six studies provided essential amino acids (EAAs) either alone [38, 41] or EAA-enriched energy-type NS [9, 27, 32, 37]. Three studies supplemented whey protein [23, 26, 40] and one studies supplemented β-hydroxy β-methylbutyrate [21]. In five studies, creatine was consumed either alone [42, 43, 45] or in combination with coenzyme QTer® [25, 35]. There studies used vitamin D3 alone [28, 36, 39], and there studies used vitamin B12 [29], polyunsaturated fatty acids [44], or magnesium citrate as supplements [20], respectively. In two studies, nutritional antioxidant supplements were used [24, 30]. In all but 1 trial the NS was given daily; in the 1 study it was consumed 100,000 IU of vitamin D per month [39]. A total of 16 studies included pulmonary rehabilitation throughout their study periods, ranging from 2 to 7 sessions per week [9, 22, 24, 27, 29, 32, 34, 37, 39–46].

### Quality assessment

**Fig. 2:**
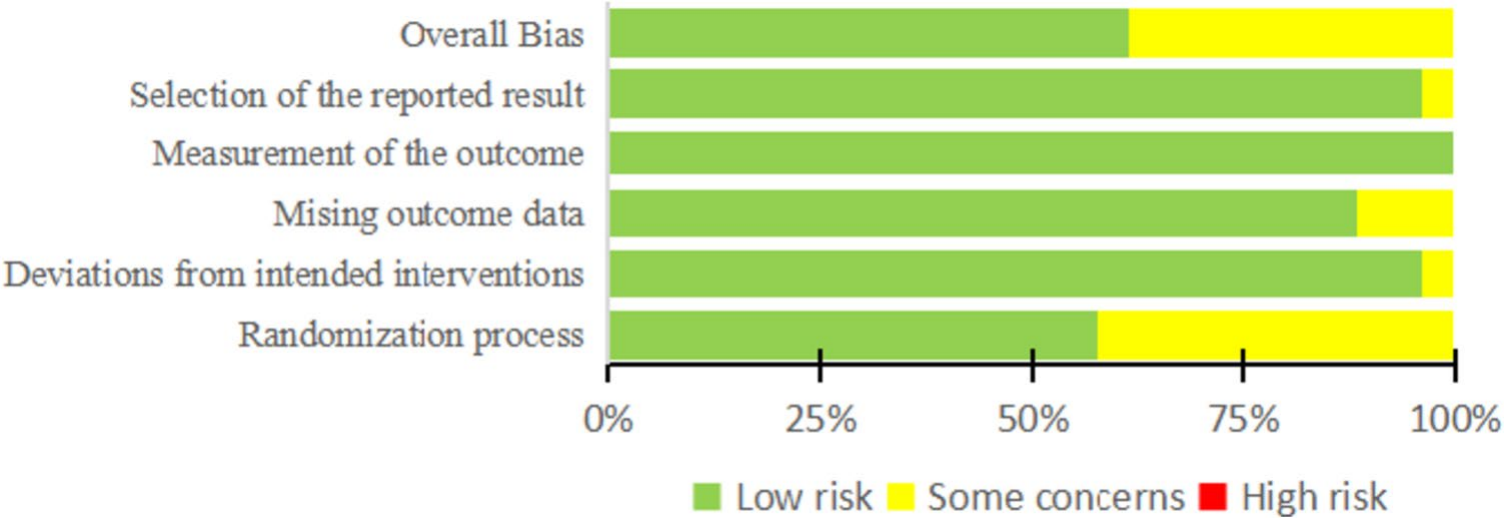
Risk of bias graph. Assessment of the risk-of-bias for each item shows as a percentage across all included studies based on the Cochrane risk-of-bias 2 tools

Figure 2 summarized the risk of bias for the included studies, while Figure S1 listed the risk of bias for each study. Eight studies raised some concerns about the randomization process due to a lack of information regarding randomization and concealment procedures [9, 33, 35, 40, 41, 44, 45, 47]. One study had some concerns arising from absence of a concealment procedure and inadequate follow-up [30]. One study had some concerns because there was no concealment procedure and no placebo preparation, which compromised the blinding of the control group [32]. One study had some concerns arising from absence of information on dropout rate [38].

### Effects of NS on body weight (BW) and BMI

**Fig. 3:**
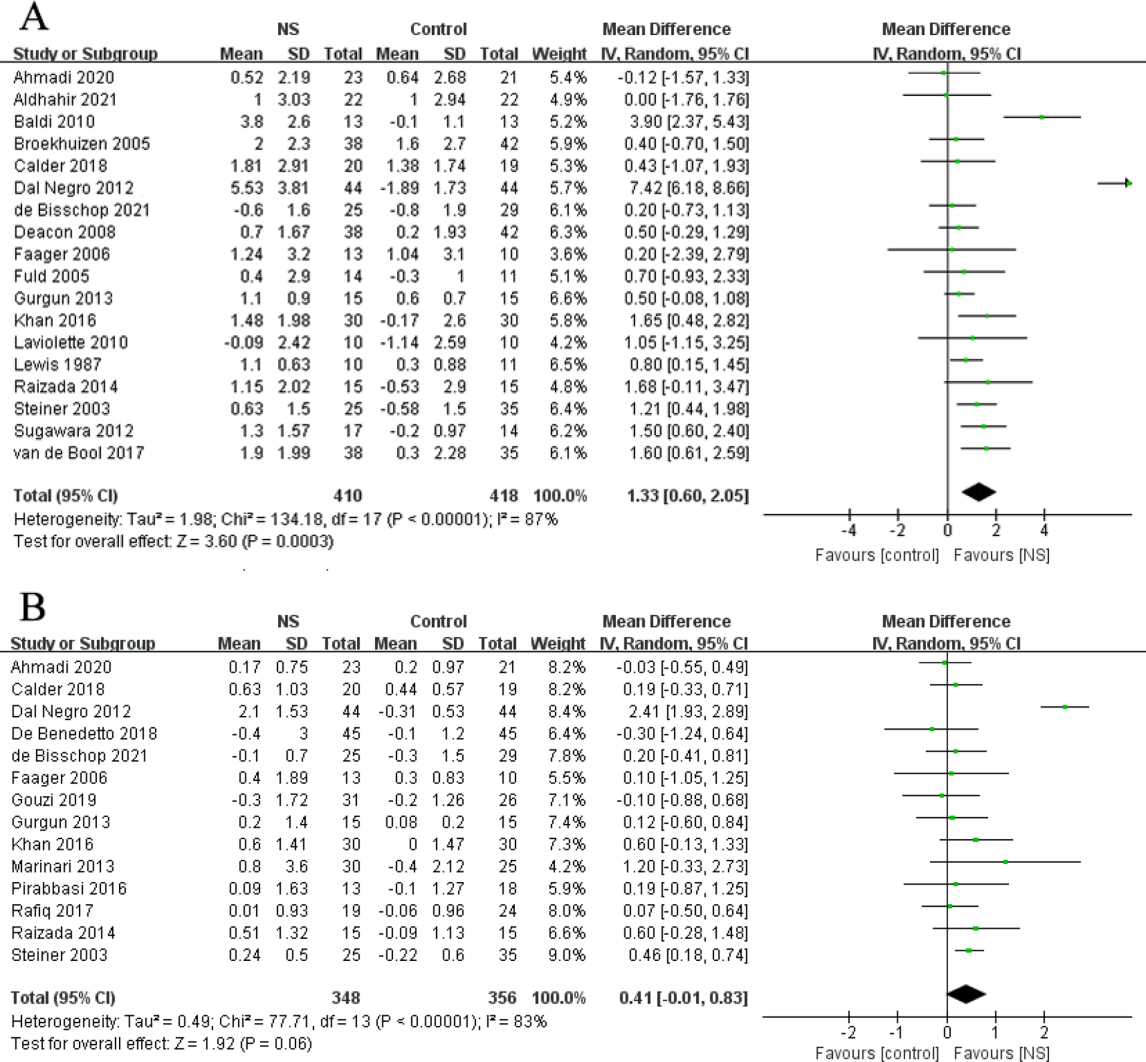
Forest plot of meta-analysis results from the effect of nutrient supplements on changes in body weight (**A**) and body mass index (**B**) in people with chronic obstructive pulmonary disease

Meta-analysis of mean differences in BW included eighteen studies with 410 NS participants and 418 control participants. In Fig. 3A, NS showed a positive effect on improving BW (MD: 1.33 kg; 95% CI: 0.60, 2.05 kg; P = 0.0003; I^2^ = 87%). Analyzing sensitivity revealed similar results.

BMI was compared between NS groups and control groups in fourteen studies. As shown in Fig. 3B, BMI was close to statistical significance with a small effect size (MD: 0.41 kg/m^2^; 95% CI: -0.01, 0.83 kg/m^2^; P = 0.06; I^2^ = 83%). However, sensitivity analyses found no heterogeneity among studies after the exclusion of Dal Negro 2012 study [38] (I^2^ = 0%), with a significant increase in BMI (MD: 0.27 kg/m^2^; 95% CI: 0.10, 0.43 kg/m^2^; P = 0.002; I^2^ = 0%) in favor of NS.

### Effects of NS on muscle mass

**Fig. 4:**
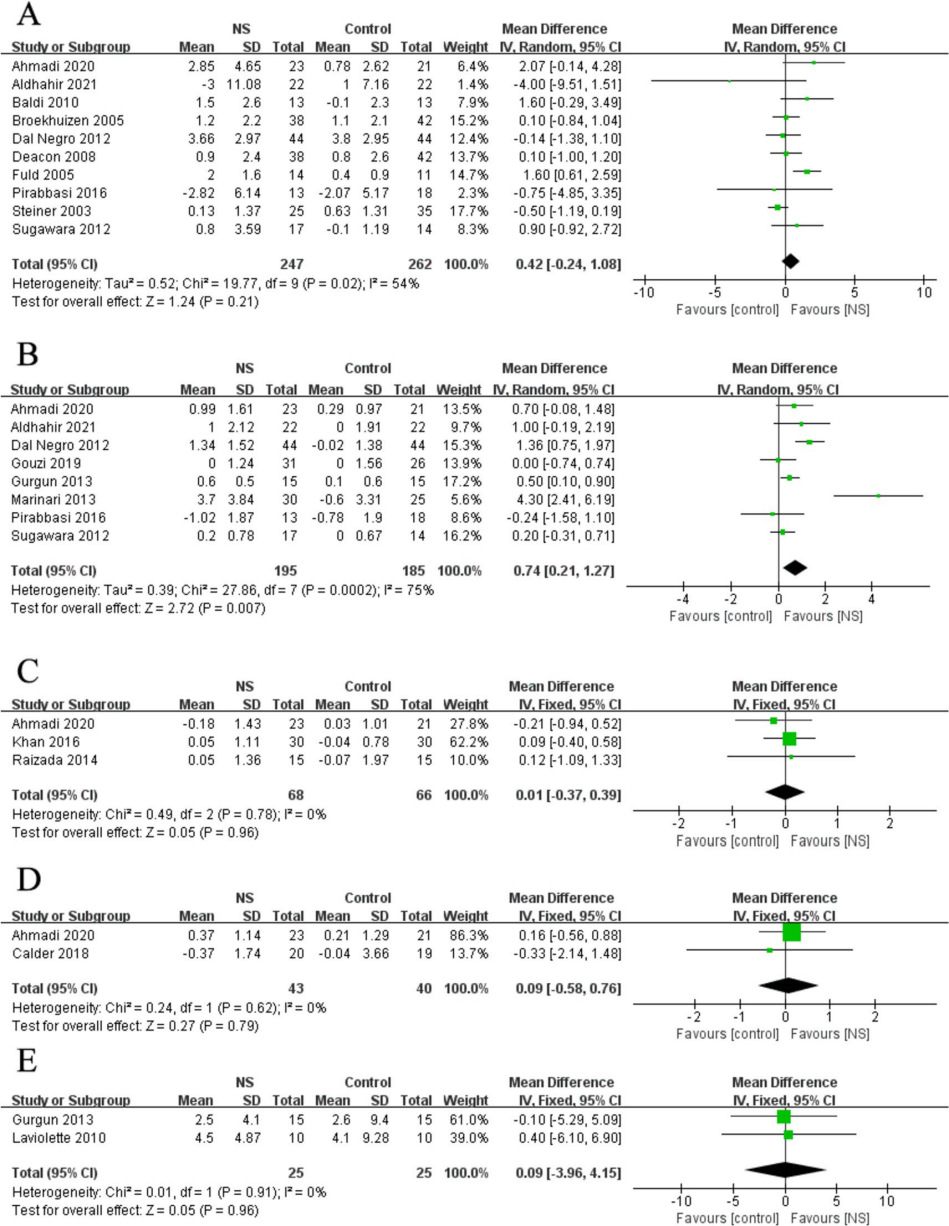
Forest plot of meta-analysis results from the effect of nutrient supplements on changes in fat-free mass (**A**), fat-free mass index (**B**), arm circumference (**C**), calf circumference (**D**), and mid-thigh cross-sectional area (**E**) in people with chronic obstructive pulmonary disease

There were seventeen RCTs that provided data to reflect between-group difference in muscle mass, measured by FFM, fat-free mass index (FFMI), arm circumference (AC), calf circumference (CC), and mid-thigh cross-sectional area (mid-thigh CSA) (Fig. 4). The meta-analysis found a significant increase in FFMI (MD: 0.74 kg/m^2^; 95% CI: 0.21, 1.27 kg/m^2^; P = 0.007; I^2^ = 75%) following NS treatment, but no significant effect on FFM (MD: 0.42 kg; 95% CI: − 0.24, 1.08 kg; P = 0.21; I^2^ = 54%), AC (MD: 0.01 cm; 95% CI: − 0.37, 0.39 cm; P = 0.96; I^2^ = 0%), CC (MD: 0.09 cm; 95% CI: − 0.58, 0.76 cm; P = 0.79; I^2^ = 0%), and mid-thigh CSA (MD: 0.09 cm^2^; 95% CI: − 3.96, 4.15 cm^2^; P = 0.96; I^2^ = 0%) following NS treatment compared to the control group.

In the sensitivity analyses, the heterogeneity was reduced after removing Marinari 2013 results [35] from the pooled estimate of FFMI, but the statistical significance was not changed. Other outcomes of muscle mass were not affected by sensitivity analyses.

### Effects of NS on muscle strength

**Fig. 5:**
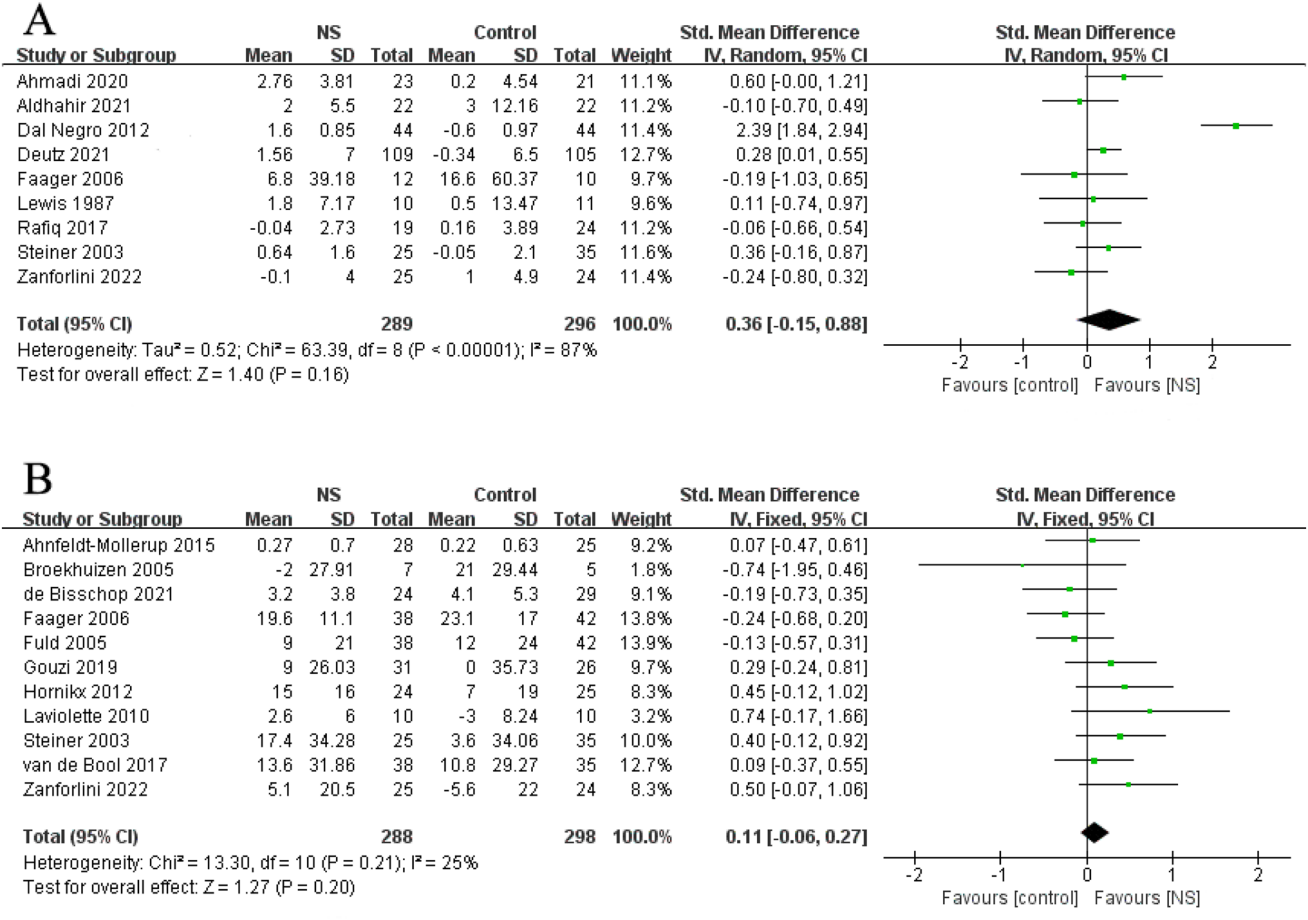
Forest plot of meta-analysis results from the effect of nutrient supplements on changes in handgrip strength (**A**) and quadriceps muscle strength (**B**) in people with chronic obstructive pulmonary disease

The effects of NS on the muscle strength of people with COPD were determined by measuring handgrip strength (HGS) and quadriceps muscle strength (QMS). There were non-significant effects on HGS and QMS with SMDs of 0.36 (95% CI: − 0.15, 0.88; P = 0.16; I^2^ = 87%) and 0.11 (95% CI: − 0.06, 0.27; P = 0.20; I^2^ = 25%), respectively, in meta-analyses (Fig. 5).

The exclusion of Dal Negro 2012 study [38] resulted in a decrease in heterogeneity among studies (I^2^ = 3%), but the statistical significance was not changed in HGS (SMD: 0.17; 95% CI: -0.02, 0.35; P = 0.07; I^2^ = 3%). In addition, no study changed QMS outcomes based on sensitivity analyses.

### Effects of NS on physical performance

**Fig. 6:**
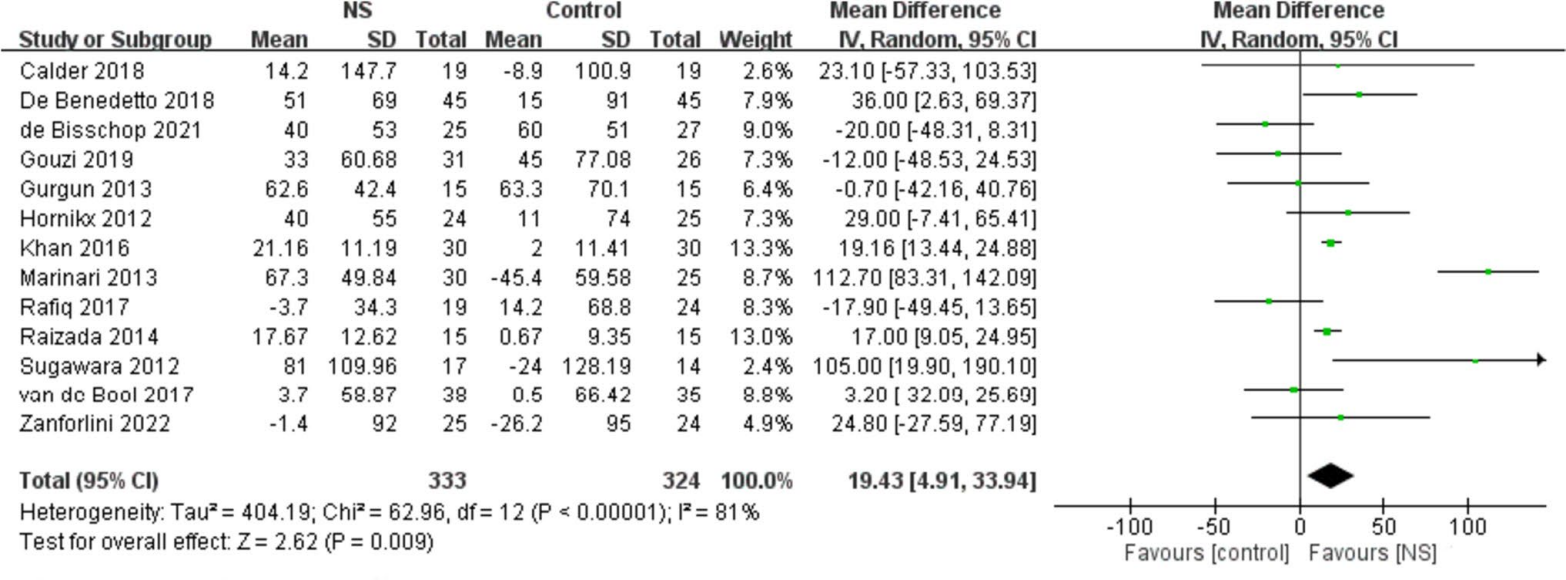
Forest plot of meta-analysis results from the effect of nutrient supplements on changes in 6-min walk test in people with chronic obstructive pulmonary disease

Several tests were used to estimate the treatment effects of NS on physical performance: 6-min walk test (6-MWT), physical activity level (PAL), incremental shuttle walk test (ISWT), endurance shuttle walk test (ESWT), short physical performance battery (SPPB), five-repetition sit-to-stand test (STS5), and cycle endurance time (CET) (Fig. 6 and Fig. S2). NS showed significant benefits to 6-MWT (MD: 19.43 m; 95% CI: 4.91, 33.94 m; P = 0.009; I^2^ = 81%) (Fig. 6). Furthermore, no study changed physical performance outcomes based on sensitivity analyses.

### Subgroup analyses

Stratified analyses showed that intervention durations appeared to have no effect on the role of NS in improving sarcopenia (Figs. S3–S9). There was no significant change in the effects of NS on sarcopenia regardless of whether NS was combined with pulmonary rehabilitation (Figs. S10–S16). Contrary to this, 6-MWT appeared to have a benefit in the absence of pulmonary rehabilitation, although it was not statistically significant (P = 0.08; Fig.  S16). Subgroup analyses comparing the types of NS revealed no significant differences (Figs. S17–S21).

## Discussion

In this meta-analysis, NS was evaluated for its efficacy in treating patients with COPD predisposed to sarcopenia. Compared with control (usual care or placebo supplements), NS positively affected BW, FFMI, 6-MWT, PAL, and STS5 in patients with COPD. In contrast, NS did not improve muscle strength (HGS and QMS) in patients with COPD. In addition, insufficient studies were included in the subgroup analyses to derive the effect of intervention durations, specific NS types, or combined training on the role of NS in improving sarcopenia.

Systematic reviews have found inconsistent results regarding nutritional support in patients with COPD. In earlier studies, nutritional support failed to demonstrate benefits for patients with COPD [48–51]. Although muscle strength was not improved by NS, the overall effects were consistent with the improvements in clinically relevant outcomes observed in recent meta-analysis studies of nutritional interventions [13, 14, 52, 53]. According to these findings, nutritional support is crucial in the treatment of patients with COPD.

Patients with COPD may respond satisfactorily to NS, resulting in augmentation of BW and muscle mass during the intervention. Based on an epidemiologic study involving 1898 participants, Vestbo et al. reported an increased mortality rate among patients with COPD having low BMI or FFMI [54]. Reduced nutrient intake contributed to weight loss or muscle loss in patients with COPD [55]. Collins et al. reported that NS can improve the nutritional status of patients and overcome energy imbalance [53]. No response to NS was noted in AC, CC, and midthigh CAS, suggesting that patients with COPD may have a balanced distribution of increased FFM throughout their bodies.

HGS is used extensively in studies focusing on sarcopenia because of its practicality and low cost in the clinical setting [56]. The HGS results of this review differed from those of previous systematic reviews regarding NS for patients with COPD. According to a previous study, the NS group had improved HGS compared with the control group when dietary counseling and enteral nutrition were included as interventions [13]. In addition, a positive effect of nutritional support was found in the pooled estimates of HGS expressed as percentage changes [14, 53]. In a 4-year prospective study of 3018 older adults, the loss of HGS rapidly outpaced the loss of muscle mass, suggesting that the positive effects of NS on HGS may require longer-term intervention in patients with COPD [57].

Patients with COPD showed significant improvements in 6-MWT, PAL, and STS5 levels after NS intervention. NS was effective in treating patients with COPD; however, previous meta-analyses excluded physical performance as a measurement outcome [13, 14, 52, 53]. In people with chronic respiratory disease, 6-MWT, ISWT, and ESWT are commonly used to assess exercise capacity [58]. An association was found between 6-MWT and mortality, and ISWT was a predictor of survival. However, fewer studies have examined ESWT in patients with COPD [58]. The minimal important differences for 6-MWT, ISWT, and ESWT were 30 m, 47.5 m, and 45–85 s, respectively [34, 58]. 6-MWT significantly improved after NS; however, ISWT and ESWT require further studies. Patients’ daily habits have a major effect on their PAL, which represents their average number of steps per day [38]. Therefore, this result should be interpreted with caution because of its limited accuracy. In addition, the SPPB or STS5 results were based on a meta-analysis of only one study; therefore, well-designed RCTs are needed to examine the effects of NS on SPPB or STS5. In patients with COPD, CET is used to assess exercise tolerance and is not interchangeable with 6-MWT [59]. Interestingly, patients with rehabilitation training reported no significant improvement in CET after 3 times/week endurance and resistance training [27, 29, 40]; however, patients with NS without rehabilitation training showed the greatest improvement [29]. These findings suggest that posttraining fatigue may affect outcome measurements or that dietary supplements fail to counteract exercise exertion.

In the subgroup analysis, no difference was found between long-term (≥ 12 weeks) and short-term (< 12 weeks) intervention in improving sarcopenia in patients with COPD. Surprisingly, NS combined with pulmonary rehabilitation reduced 6-MWT more than presented a greater benefit, suggesting that posttraining fatigue may affect outcome measurements or dietary supplements fail to counteract exercise exertion. The combined intervention also reduced mobility in malnourished older adults compared with NS alone [60]. Ongoing calorie supplementation through NS significantly improves the BW of malnourished patients with COPD and their quality of life [61]. On the basis of our findings, the study failed to present a difference in the role of NS providing energy nutrients versus NS providing nonenergy nutrients for treating sarcopenia in patients with COPD.

Patients with COPD have different degrees of muscle atrophy (i.e., secondary sarcopenia) because of advanced age, less physical activity, hypoxemia, systemic inflammation, reduced nutritional intake, and glucocorticoids [55, 62]. Sarcopenia is a key extrapulmonary feature of COPD and is characterized by reductions in muscle quality and quantity [4, 5]. Patients with COPD and sarcopenia have impaired physical function, increased degrees of dyspnea, poor prognosis, and a high risk of exacerbation or death [5, 6]. It can significantly improve the health-related quality of life of patients with COPD by reducing the risk of sarcopenia. Malnutrition is a correctable risk factor for sarcopenia. An NS that targets nutrient provision can correct a reduction in nutritional intake. Evidence containing nearly half of the low-to-moderate quality RCTs showed that NS could improve body composition and physical performance in patients with COPD. This study highlights the need for future high-quality RCTs that use standardized outcomes of sarcopenia when exploring the role of NS in treating sarcopenia.

Several limitations should be considered in this study. First, although the NS regimens were homogeneous among the included studies, some variations were noted among NS regimens; therefore, determining the effect of a specific type of NS on sarcopenia was difficult, and the clinical heterogeneity inevitably affected the results. Second, the subgroup analysis lacked statistical power. Fewer than three RCTs were included in subgroup analyses stratified by NS type, suggesting that such analyses were not sufficiently powerful to detect changes. Third, the severity of COPD could not be classified. Indeed, most of the included studies enrolled patients with moderate-to-severe COPD; however, the use of the criterion of FEV_1_ less than the percentage of predicted values prevented us from categorizing the whole sample. Finally, sensitivity analyses were conducted to rule out possible effects; however, heterogeneity related to race and measurement tools was not negligible, affecting the reliability of the evidence.

## Conclusion

In this systematic review and meta-analysis, NS was associated with weight gain and increases in FFMI, 6-MWT, PAL, and STS5, whereas increases in muscle strength may require longer NS interventions. A subgroup analysis revealed a downward trend in 6-MWT following combined pulmonary rehabilitation, indicating a potential effect of posttraining fatigue or increased energy expenditure after exercise. The effect of NS types on sarcopenia was inconclusive because of the lack of trials. Further research on intervention durations, NS types, or NS combined training in populations with COPD is required to gain insight into their effects on sarcopenia. Based on the available evidence, NS is a feasible treatment option for COPD-related sarcopenia.

### Supplementary Information

Below is the link to the electronic supplementary material.

Supplementary file1 (PDF 1078 KB)

## Data Availability

The data used to support the findings of this study have been included in this article.
