# Supplementary material for: Systematic review and meta-analysis of nutrient supplements for treating sarcopenia in people with chronic obstructive pulmonary disease
Source: Aging Clin Exp Res. 2024 Mar 14;36(1):69. doi: 10.1007/s40520-024-02722-w (PMC10940388; doi:10.1007/s40520-024-02722-w)
Supplement: Supplementary file 1 — Supplementary file1 (PDF 1078 KB) [file 40520_2024_2722_MOESM1_ESM.pdf]

Supplementary data

In our study, the comprehensive search strategy is detailed in Table S1. Regarding the quality assessment of the articles, Figure S1 presents the risk of bias evaluation for each included study.

For the assessment of nutrient supplements (NS) on physical performance, the effects of NS treatment were evaluated using various tests. These included the 6-minute walk test (6-MWT), physical activity level (PAL), incremental shuttle walk test (ISWT), endurance shuttle walk test (ESWT), short physical performance battery (SPPB), five-repetition sit-to-stand test (STS5), and cycle endurance time (CET), as illustrated in Figure S2.

In the conducted subgroup analyses, stratification by intervention periods, as displayed in Figures S3 to S9, revealed that NS with a longer intervention duration (≥ 12 weeks) resulted in more significant improvements in both body weight (P=0.04; Figure S3) and quadriceps muscle strength (P=0.01; Figure S8) compared to NS with shorter intervention periods (< 12 weeks). Interestingly, the impact of NS on sarcopenia did not show significant changes irrespective of the combination with pulmonary rehabilitation, as illustrated in Figures S10 to S16. In contrast, the 6-minute walk test (6-MWT) demonstrated potential benefits when not combined with pulmonary rehabilitation, though this finding was not statistically significant (P=0.18; Figure S16). Furthermore, subgroup analyses comparing different types of NS did not reveal any significant differences, as shown in Figures S17 to S21.

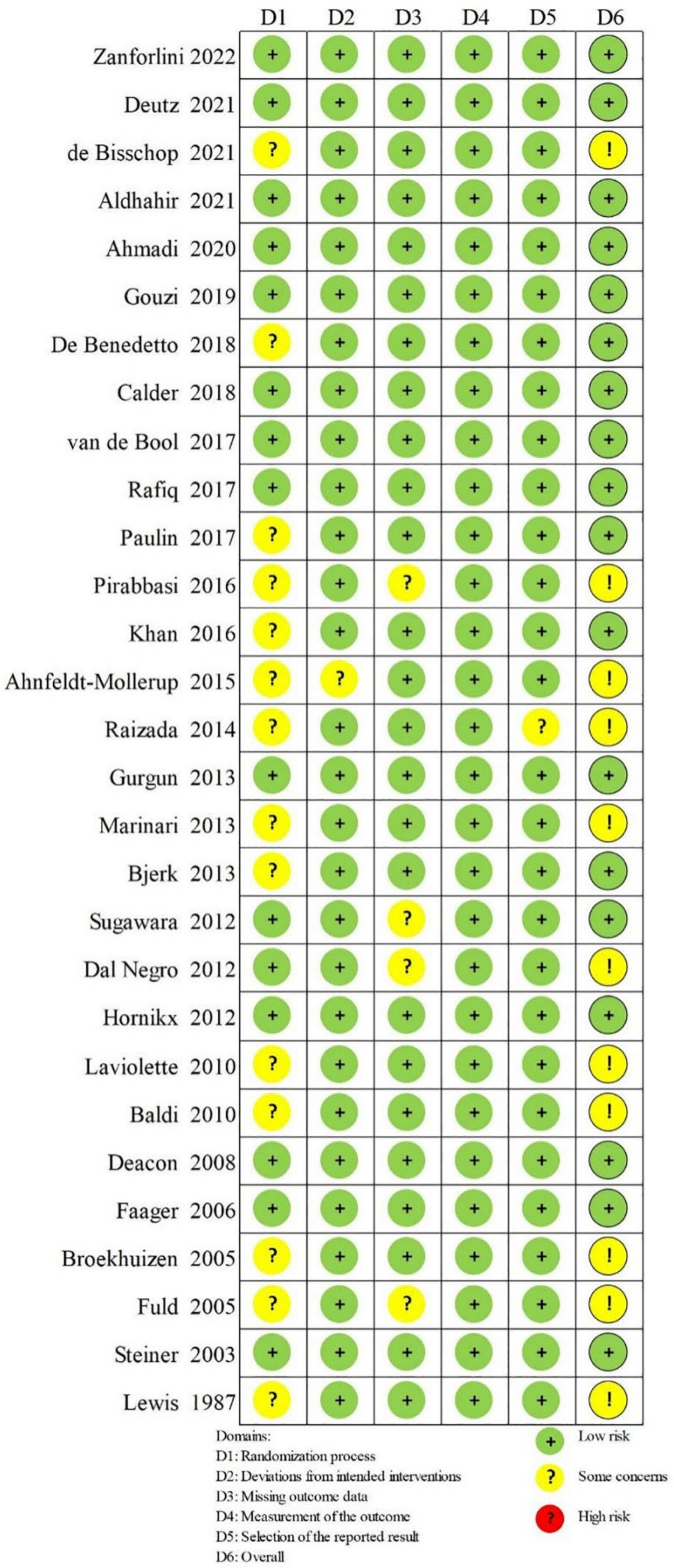

Figure S1. Risk of bias graph. Assessment of the risk-of-bias for each study is based on the Cochrane risk-of-bias 2 tools.

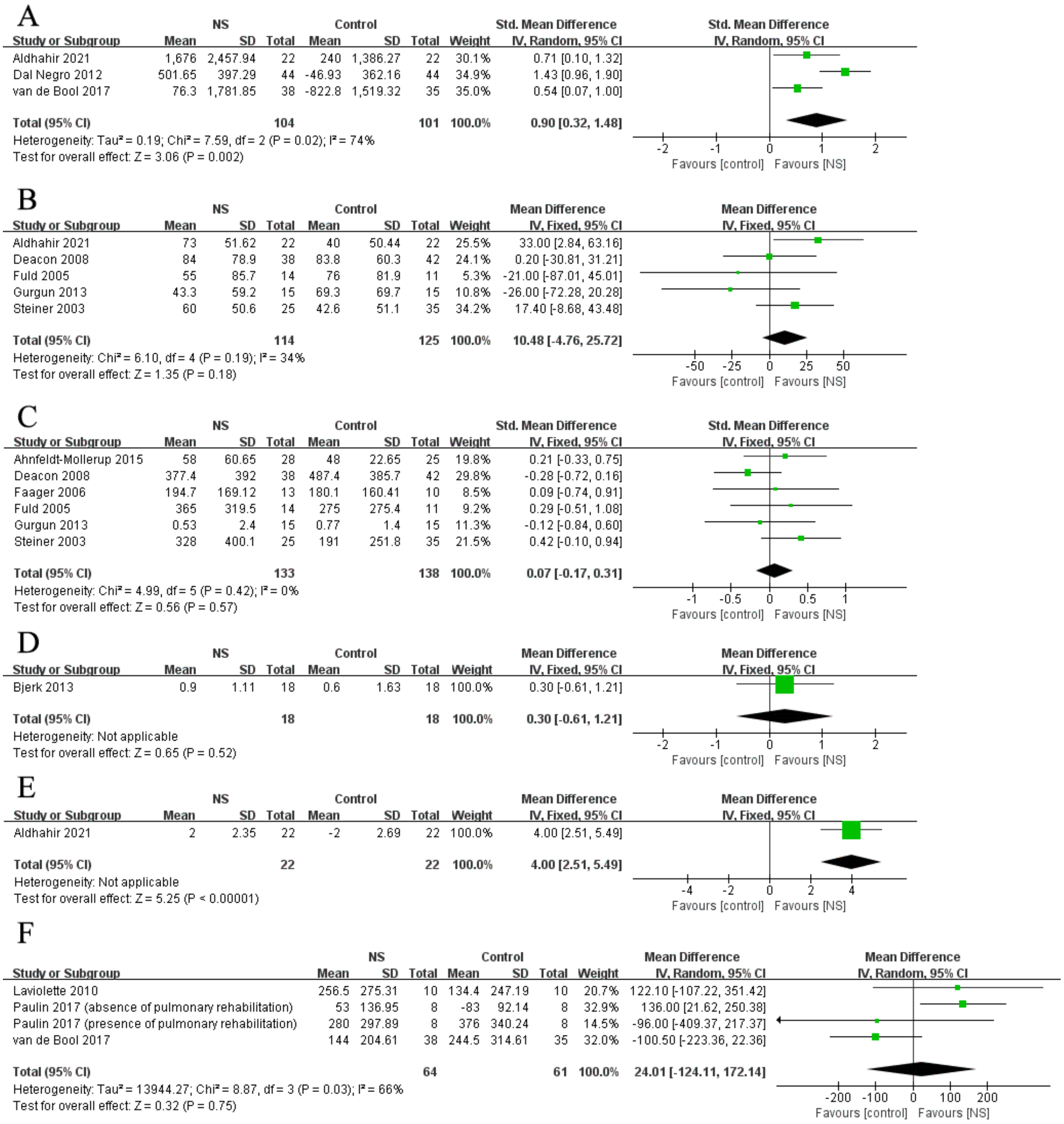

Figure S2. Forest plot of meta-analysis results from the effect of nutrient supplements on changes in physical activity level (A), incremental shuttle walk test (B), endurance shuttle walk test (C), short physical performance battery (D), five-repetition sit-to-stand test (E), cycle endurance time (F) in people with chronic obstructive pulmonary disease.

Study or Subgroup	NS Mean	NS SD	NS Total	Control Mean	Control SD	Control Total	Weight	Mean Difference IV, Random, 95% CI
**3.1 body weight (kg), short (<12wk)**								
Ahmadi 2020	0.52	2.19	23	0.64	2.68	21	4.6%	-0.12 [-1.57, 1.33]
Aldhahir 2021	1	3.03	22	1	2.94	22	4.1%	0.00 [-1.76, 1.76]
Broekhuizen 2005	2	2.3	38	1.6	2.7	42	5.0%	0.40 [-0.70, 1.50]
Dal Negro 2012 (4 wk)	1.93	2.4	44	-1.1	1.57	44	5.4%	3.03 [2.18, 3.88]
de Bisschop 2021	-0.6	1.6	25	-0.8	1.9	29	5.3%	0.20 [-0.73, 1.13]
Deacon 2008	0.7	1.67	38	0.2	1.93	42	5.4%	0.50 [-0.29, 1.29]
Faager 2006	1.24	3.2	13	1.04	3.1	10	3.1%	0.20 [-2.39, 2.79]
Gurgun 2013	1.1	0.9	15	0.6	0.7	15	5.6%	0.50 [-0.08, 1.08]
Laviolette 2010 (8 wk)	0.39	2.54	10	0.35	1.58	10	4.0%	0.04 [-1.81, 1.89]
Lewis 1987	1.1	0.63	10	0.3	0.88	11	5.6%	0.80 [0.15, 1.45]
Raizada 2014	1.15	2.02	15	-0.53	2.9	15	4.1%	1.68 [-0.11, 3.47]
Steiner 2003	0.63	1.5	25	-0.58	1.5	35	5.4%	1.21 [0.44, 1.98]
**Subtotal (95% CI)**			**278**			**296**	**57.5%**	**0.79 [0.26, 1.33]**
Heterogeneity: Tau² = 0.54; Chi² = 35.37, df = 11 (P = 0.0002); I² = 69%								
Test for overall effect: Z = 2.90 (P = 0.004)								
**3.2 body weight (kg), long (≥12wk)**								
Baldi 2010	3.8	2.6	13	-0.1	1.1	13	4.7%	3.90 [2.37, 5.43]
Calder 2018	1.81	2.91	20	1.38	1.74	19	4.8%	0.43 [-1.07, 1.93]
Dal Negro 2012 (12 wk)	5.53	3.81	44	-1.89	1.73	44	5.1%	7.42 [6.18, 8.66]
Fuld 2005	0.4	2.9	14	-0.3	1	11	4.6%	0.70 [-0.93, 2.33]
Khan 2016	1.48	1.98	30	-0.17	2.6	30	5.2%	1.65 [0.48, 2.82]
Laviolette 2010 (16 wk)	-0.09	2.42	10	-1.14	2.59	10	3.8%	1.05 [-1.15, 3.25]
Sugawara 2012	1.3	1.57	17	-0.2	0.97	14	5.6%	1.50 [0.60, 2.40]
van de Bool 2017	1.9	1.99	38	0.3	2.28	35	5.5%	1.60 [0.61, 2.59]
**Subtotal (95% CI)**			**186**			**176**	**39.3%**	**2.31 [0.70, 3.92]**
Heterogeneity: Tau² = 4.86; Chi² = 87.12, df = 7 (P < 0.00001); I² = 92%								
Test for overall effect: Z = 2.81 (P = 0.005)								
**Total (95% CI)**			**464**			**472**	**100.0%**	**1.37 [0.67, 2.06]**
Heterogeneity: Tau² = 2.05; Chi² = 153.73, df = 19 (P < 0.00001); I² = 88%								
Test for overall effect: Z = 3.86 (P = 0.0001)								
Test for subgroup differences: Chi² = 3.06, df = 1 (P = 0.08), I² = 67.4%								

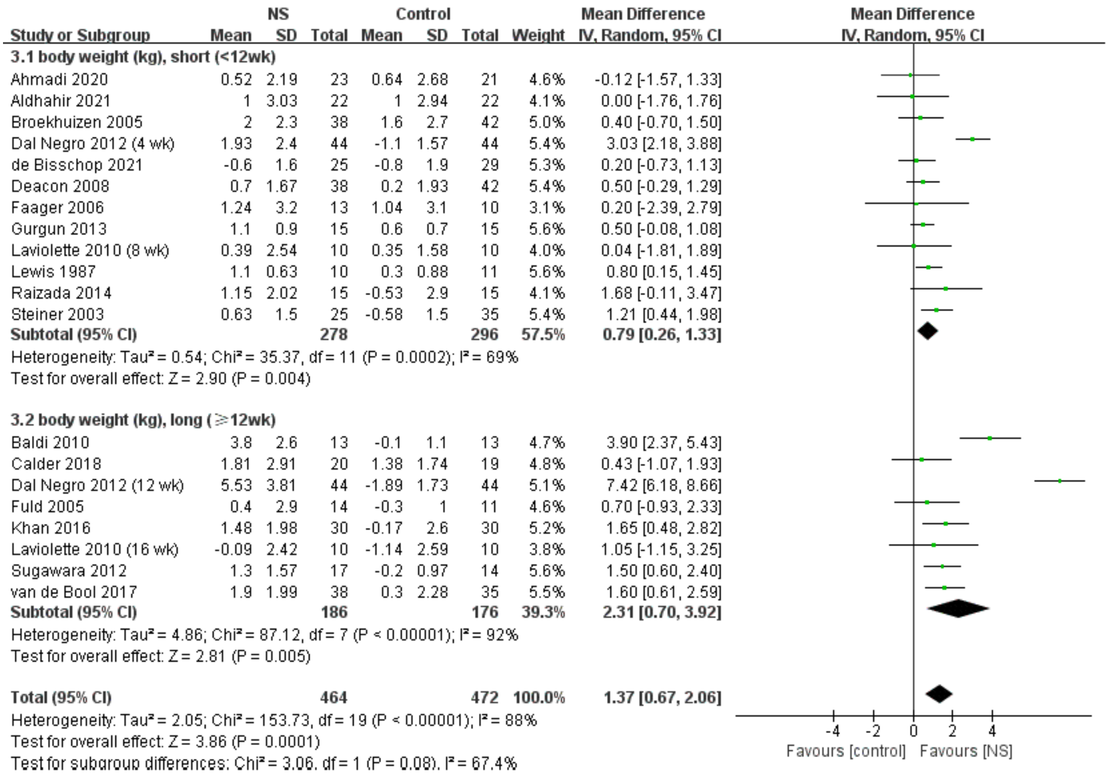

Figure S3. Forest plot of subgroup analysis results from body weight changes in response to nutrient supplements based on different intervention periods in people with chronic obstructive pulmonary disease.

	NS			Control				Mean Difference
Study or Subgroup	Mean	SD	Total	Mean	SD	Total	Weight	IV, Random, 95% CI
**4.1 body mass index (kg/m^2), short (<12wk)**								
Ahmadi 2020	0.17	0.75	23	0.2	0.97	21	7.7%	-0.03 [-0.55, 0.49]
Dal Negro 2012 (4 wk)	0.66	1.23	44	-0.21	0.54	44	8.1%	0.87 [0.47, 1.27]
De Benedetto 2018	-0.4	3	45	-0.1	1.2	45	5.8%	-0.30 [-1.24, 0.64]
de Bisschop 2021	-0.1	0.7	25	-0.3	1.5	29	7.2%	0.20 [-0.41, 0.81]
Faager 2006	0.4	1.89	13	0.3	0.83	10	4.9%	0.10 [-1.05, 1.25]
Gouzi 2019	-0.3	1.72	31	-0.2	1.26	26	6.5%	-0.10 [-0.88, 0.68]
Gurgun 2013	0.2	1.4	15	0.08	0.2	15	6.8%	0.12 [-0.60, 0.84]
Marinari 2013	0.8	3.6	30	-0.4	2.12	25	3.7%	1.20 [-0.33, 2.73]
Raizada 2014	0.51	1.32	15	-0.09	1.13	15	6.0%	0.60 [-0.28, 1.48]
Steiner 2003	0.24	0.5	25	-0.22	0.6	35	8.5%	0.46 [0.18, 0.74]
**Subtotal (95% CI)**			**266**			**265**	**65.2%**	**0.33 [0.08, 0.58]**
Heterogeneity: Tau² = 0.06; Chi² = 14.45, df = 9 (P = 0.11); I² = 38%								
Test for overall effect: Z = 2.55 (P = 0.01)								
**4.2 body mass index (kg/m^2), long (≥12wk)**								
Calder 2018	0.63	1.03	20	0.44	0.57	19	7.6%	0.19 [-0.33, 0.71]
Dal Negro 2012 (12 wk)	2.1	1.53	44	-0.31	0.53	44	7.8%	2.41 [1.93, 2.89]
Khan 2016	0.6	1.41	30	0	1.47	30	6.7%	0.60 [-0.13, 1.33]
Pirabbasi 2016	0.09	1.63	13	-0.1	1.27	18	5.3%	0.19 [-0.87, 1.25]
Rafiq 2017	0.01	0.93	19	-0.06	0.96	24	7.4%	0.07 [-0.50, 0.64]
**Subtotal (95% CI)**			**126**			**135**	**34.8%**	**0.71 [-0.33, 1.75]**
Heterogeneity: Tau² = 1.28; Chi² = 56.02, df = 4 (P < 0.00001); I² = 93%								
Test for overall effect: Z = 1.34 (P = 0.18)								
**Total (95% CI)**			**392**			**400**	**100.0%**	**0.45 [0.07, 0.83]**
Heterogeneity: Tau² = 0.43; Chi² = 80.62, df = 14 (P < 0.00001); I² = 83%								
Test for overall effect: Z = 2.31 (P = 0.02)								
Test for subgroup differences: Chi² = 0.49, df = 1 (P = 0.48), I² = 0%								

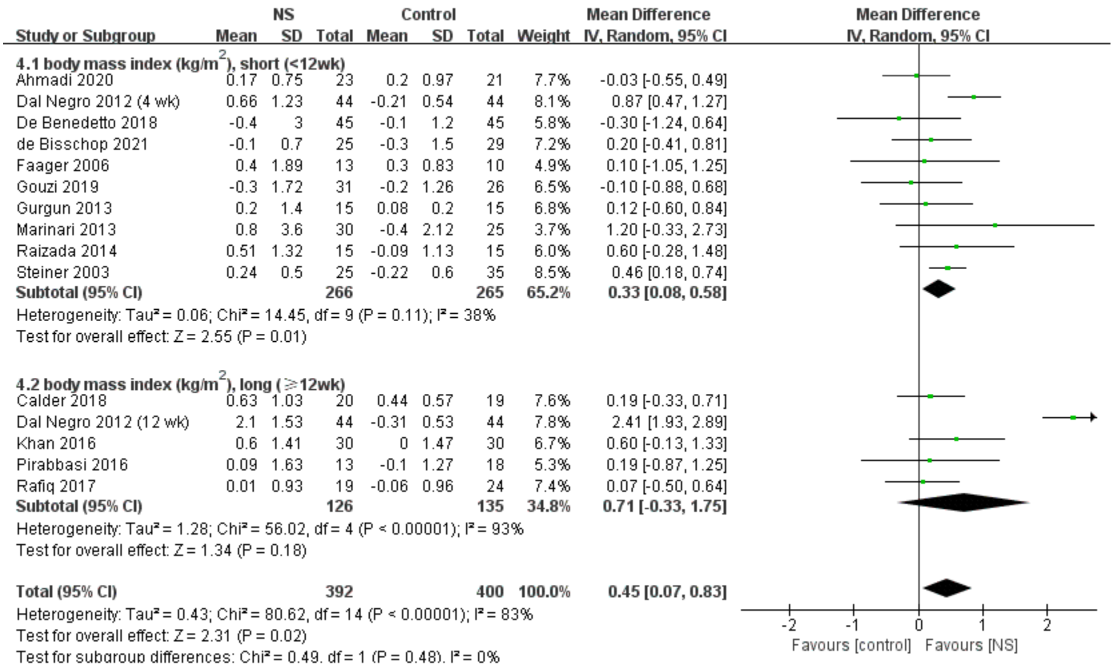

Figure S4. Forest plot of subgroup analysis results from body mass index changes in response to nutrient supplements based on different intervention periods in people with chronic obstructive pulmonary disease.

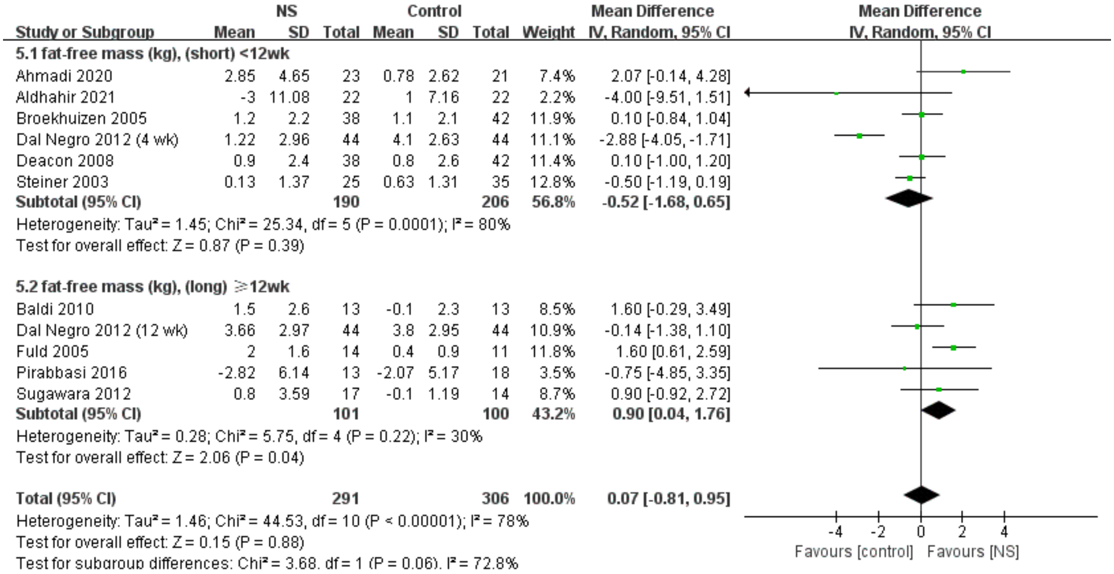

Study or Subgroup	NS Mean	NS SD	NS Total	Control Mean	Control SD	Control Total	Weight	Mean Difference IV, Random, 95% CI
**5.1 fat-free mass (kg), (short) <12wk**								
Ahmadi 2020	2.85	4.65	23	0.78	2.62	21	7.4%	2.07 [-0.14, 4.28]
Aldhahir 2021	-3	11.08	22	1	7.16	22	2.2%	-4.00 [-9.51, 1.51]
Broekhuizen 2005	1.2	2.2	38	1.1	2.1	42	11.9%	0.10 [-0.84, 1.04]
Dal Negro 2012 (4 wk)	1.22	2.96	44	4.1	2.63	44	11.1%	-2.88 [-4.05, -1.71]
Deacon 2008	0.9	2.4	38	0.8	2.6	42	11.4%	0.10 [-1.00, 1.20]
Steiner 2003	0.13	1.37	25	0.63	1.31	35	12.8%	-0.50 [-1.19, 0.19]
**Subtotal (95% CI)**			**190**			**206**	**56.8%**	**-0.52 [-1.68, 0.65]**
Heterogeneity: Tau² = 1.45; Chi² = 25.34, df = 5 (P = 0.0001); I² = 80%								
Test for overall effect: Z = 0.87 (P = 0.39)								
**5.2 fat-free mass (kg), (long) ≥12wk**								
Baldi 2010	1.5	2.6	13	-0.1	2.3	13	8.5%	1.60 [-0.29, 3.49]
Dal Negro 2012 (12 wk)	3.66	2.97	44	3.8	2.95	44	10.9%	-0.14 [-1.38, 1.10]
Fuld 2005	2	1.6	14	0.4	0.9	11	11.8%	1.60 [0.61, 2.59]
Pirabbasi 2016	-2.82	6.14	13	-2.07	5.17	18	3.5%	-0.75 [-4.85, 3.35]
Sugawara 2012	0.8	3.59	17	-0.1	1.19	14	8.7%	0.90 [-0.92, 2.72]
**Subtotal (95% CI)**			**101**			**100**	**43.2%**	**0.90 [0.04, 1.76]**
Heterogeneity: Tau² = 0.28; Chi² = 5.75, df = 4 (P = 0.22); I² = 30%								
Test for overall effect: Z = 2.06 (P = 0.04)								
**Total (95% CI)**			**291**			**306**	**100.0%**	**0.07 [-0.81, 0.95]**
Heterogeneity: Tau² = 1.46; Chi² = 44.53, df = 10 (P < 0.00001); I² = 78%								
Test for overall effect: Z = 0.15 (P = 0.88)								
Test for subgroup differences: Chi² = 3.68, df = 1 (P = 0.06), I² = 72.8%								

Figure S5. Forest plot of subgroup analysis results from fat-free mass changes in response to nutrient supplements based on different intervention periods in people with chronic obstructive pulmonary disease.

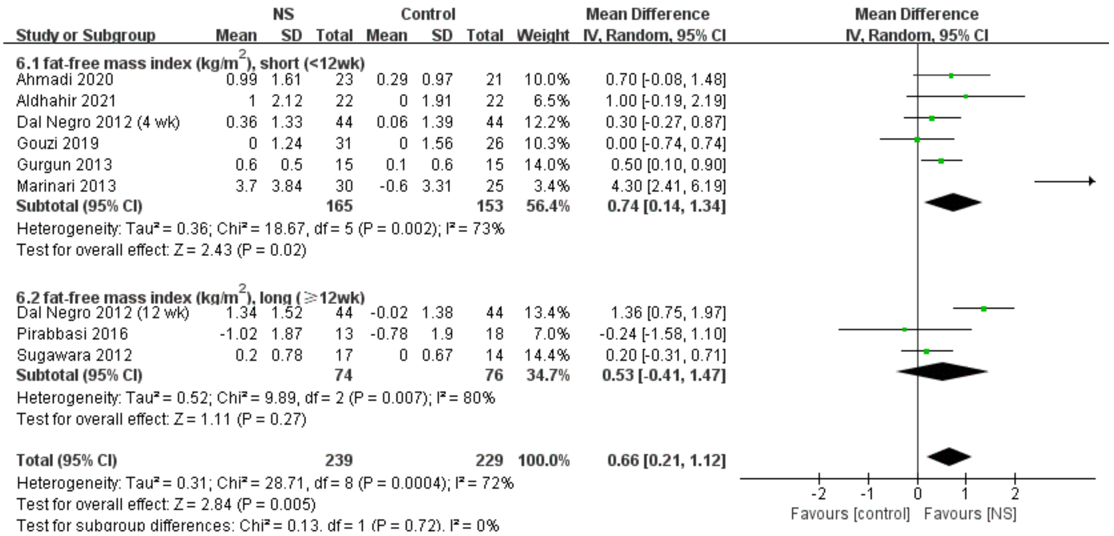

Figure S6. Forest plot of subgroup analysis results from fat-free mass index changes in response to nutrient supplements based on different intervention periods in people with chronic obstructive pulmonary disease.

	NS			Control				Std. Mean Difference
Study or Subgroup	Mean	SD	Total	Mean	SD	Total	Weight	IV, Random, 95% CI
**7.1 handgrip strength, short (<12wk)**								
Ahmadi 2020	2.76	3.81	23	0.2	4.54	21	9.0%	0.60 [-0.00, 1.21]
Aldhahir 2021	2	5.5	22	3	12.16	22	9.1%	-0.10 [-0.70, 0.49]
Dal Negro 2012 (4 wk)	0.5	0.76	44	-0.2	1.18	44	10.0%	0.70 [0.27, 1.13]
Deutz 2021	1.56	7	109	-0.34	6.5	105	10.7%	0.28 [0.01, 0.55]
Faager 2006	6.8	39.18	12	16.6	60.37	10	7.6%	-0.19 [-1.03, 0.65]
Lewis 1987	1.8	7.17	10	0.5	13.47	11	7.6%	0.11 [-0.74, 0.97]
Steiner 2003	0.64	1.6	25	-0.05	2.1	35	9.5%	0.36 [-0.16, 0.87]
**Subtotal (95% CI)**			**245**			**248**	**63.5%**	**0.32 [0.11, 0.54]**
Heterogeneity: Tau² = 0.02; Chi² = 7.51, df = 6 (P = 0.28); I² = 20%								
Test for overall effect: Z = 2.93 (P = 0.003)								
**7.2 handgrip strength, long (≥12wk)**								
Dal Negro 2012 (12 wk)	1.6	0.85	44	-0.6	0.97	44	10.2%	2.39 [1.84, 2.94]
Rafiq 2017	-0.04	2.73	19	0.16	3.89	24	9.9%	-0.06 [-0.66, 0.54]
Zanforlini 2022	-0.1	4	25	1	4.9	24	10.2%	-0.24 [-0.80, 0.32]
**Subtotal (95% CI)**			**88**			**92**	**30.3%**	**0.70 [-1.00, 2.40]**
Heterogeneity: Tau² = 2.16; Chi² = 52.77, df = 2 (P < 0.00001); I² = 96%								
Test for overall effect: Z = 0.81 (P = 0.42)								
**Total (95% CI)**			**333**			**340**	**100.0%**	**0.40 [-0.05, 0.85]**
Heterogeneity: Tau² = 0.44; Chi² = 65.22, df = 9 (P < 0.00001); I² = 86%								
Test for overall effect: Z = 1.75 (P = 0.08)								
Test for subgroup differences: Chi² = 0.19, df = 1 (P = 0.67), I² = 0%								

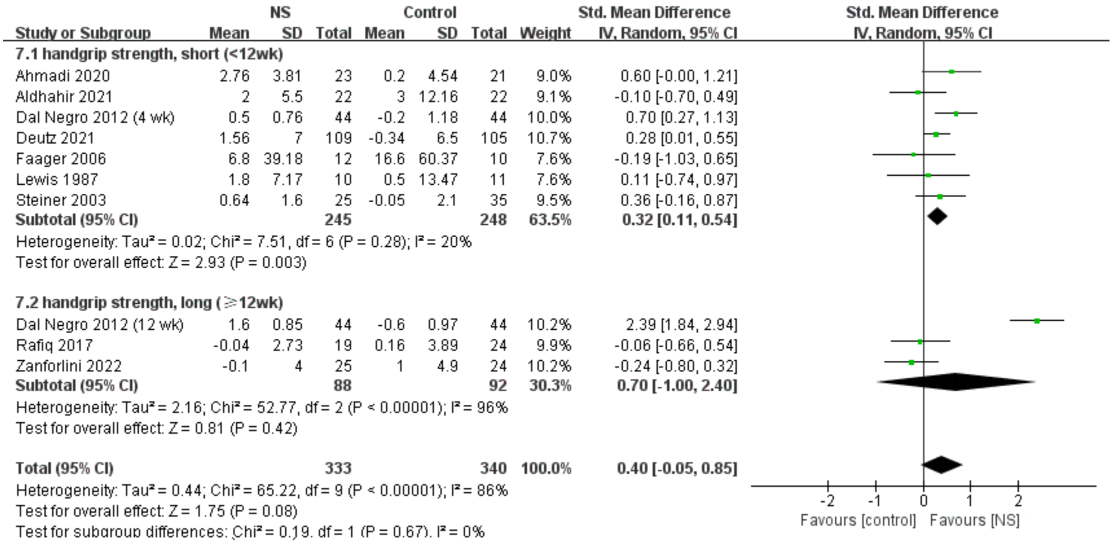

Figure S7. Forest plot of subgroup analysis results from handgrip strength changes in response to nutrient supplements based on different intervention periods in people with chronic obstructive pulmonary disease.

Study or Subgroup	NS Mean	NS SD	NS Total	Control Mean	Control SD	Control Total	Weight	Std. Mean Difference IV, Fixed, 95% CI
**8.1 quadriceps muscle strength, short (<12wk)**								
Ahnfeldt-Mollerup 2015	0.27	0.7	28	0.22	0.63	25	8.4%	0.07 [-0.47, 0.61]
Broekhuizen 2005	-2	27.91	7	21	29.44	5	1.7%	-0.74 [-1.95, 0.46]
de Bisschop 2021	3.2	3.8	24	4.1	5.3	29	8.4%	-0.19 [-0.73, 0.35]
Faager 2006	19.6	11.1	38	23.1	17	42	12.7%	-0.24 [-0.68, 0.20]
Fuld 2005	9	21	38	12	24	42	12.7%	-0.13 [-0.57, 0.31]
Gouzi 2019	9	26.03	31	0	35.73	26	9.0%	0.29 [-0.24, 0.81]
Laviolette 2010 (8 wk)	1.5	6.11	12	0.7	7.81	10	3.5%	0.11 [-0.73, 0.95]
Steiner 2003	17.4	34.28	25	3.6	34.06	35	9.1%	0.40 [-0.12, 0.92]
**Subtotal (95% CI)**			**203**			**214**	**65.5%**	**-0.00 [-0.20, 0.19]**
Heterogeneity: Chi² = 6.98, df = 7 (P = 0.43); I² = 0%								
Test for overall effect: Z = 0.05 (P = 0.96)								
**8.2 quadriceps muscle strength, long (≥12wk)**								
Hornikx 2012	15	16	24	7	19	25	8.0%	0.45 [-0.12, 1.02]
Laviolette 2010 (16 wk)	2.6	6	10	-3	8.24	10	3.1%	0.74 [-0.17, 1.66]
van de Bool 2017	13.6	31.86	38	10.8	29.27	35	12.2%	0.09 [-0.37, 0.55]
Zanforlini 2022	5.1	20.5	25	-5.6	22	24	8.0%	0.50 [-0.07, 1.06]
**Subtotal (95% CI)**			**97**			**94**	**31.3%**	**0.35 [0.06, 0.64]**
Heterogeneity: Chi² = 2.31, df = 3 (P = 0.51); I² = 0%								
Test for overall effect: Z = 2.38 (P = 0.02)								
**Total (95% CI)**			**300**			**308**	**100.0%**	**0.11 [-0.05, 0.27]**
Heterogeneity: Chi² = 13.30, df = 11 (P = 0.27); I² = 17%								
Test for overall effect: Z = 1.29 (P = 0.20)								
Test for subgroup differences: Chi² = 4.02, df = 1 (P = 0.05), I² = 75.1%								

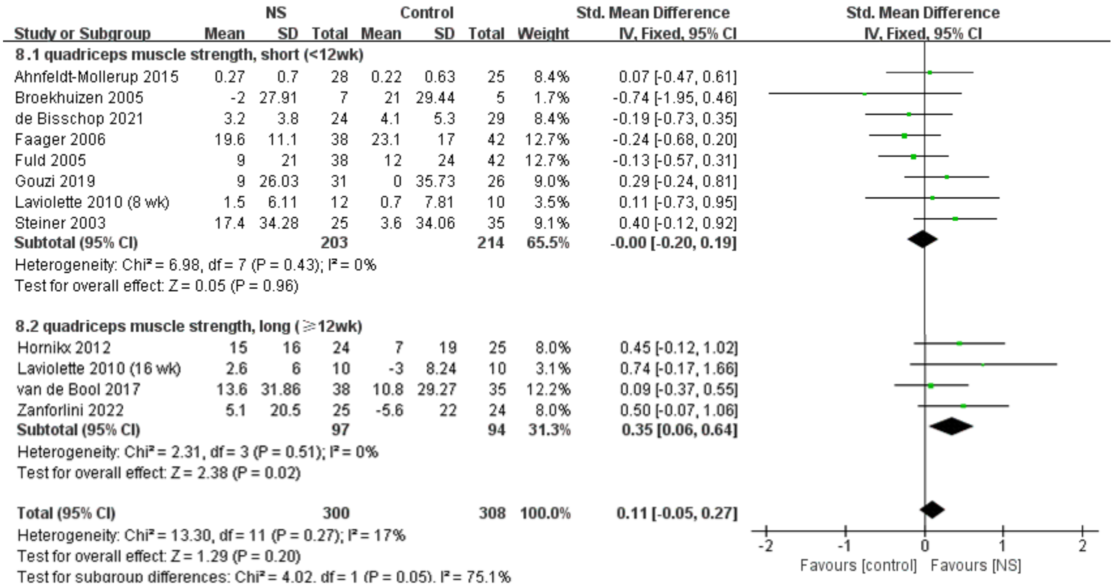

Figure S8. Forest plot of subgroup analysis results from quadriceps muscle strength changes in response to nutrient supplements based on different intervention periods in people with chronic obstructive pulmonary disease.

	NS			Control				Mean Difference
Study or Subgroup	Mean	SD	Total	Mean	SD	Total	Weight	IV, Random, 95% CI
**9.1 6-minute walk test (m), short (<12wk)**								
De Benedetto 2018	51	69	45	15	91	45	7.7%	36.00 [2.63, 69.37]
de Bisschop 2021	40	53	25	60	51	27	8.7%	-20.00 [-48.31, 8.31]
Gouzi 2019	33	60.68	31	45	77.08	26	7.1%	-12.00 [-48.53, 24.53]
Gurgun 2013	62.6	42.4	15	63.3	70.1	15	6.2%	-0.70 [-42.16, 40.76]
Marinari 2013	67.3	49.84	30	-45.4	59.58	25	8.4%	112.70 [83.31, 142.09]
Raizada 2014	17.67	12.62	15	0.67	9.35	15	12.6%	17.00 [9.05, 24.95]
**Subtotal (95% CI)**			**161**			**153**	**50.7%**	**22.72 [-10.75, 56.19]**
Heterogeneity: Tau² = 1504.43; Chi² = 51.21, df = 5 (P < 0.00001); I² = 90%								
Test for overall effect: Z = 1.33 (P = 0.18)								
**9.2 6-minute walk test (m), long (≥12wk)**								
Calder 2018	14.2	147.7	19	-8.9	100.9	19	2.6%	23.10 [-57.33, 103.53]
Hornikx 2012	40	55	24	11	74	25	7.3%	29.00 [-7.41, 65.41]
Khan 2016	21.16	11.19	30	2	11.41	30	13.3%	19.16 [13.44, 24.88]
Rafiq 2017	-3.7	34.3	19	14.2	68.8	24	8.3%	-17.90 [-49.45, 13.65]
Sugawara 2012	81	109.96	17	-24	128.19	14	2.4%	105.00 [19.90, 190.10]
van de Bool 2017	-3.7	58.87	38	-0.5	66.42	35	8.8%	-3.20 [-32.09, 25.69]
Zanforlini 2022	-1.4	92	25	-26.2	95	24	4.9%	24.80 [-27.59, 77.19]
**Subtotal (95% CI)**			**172**			**171**	**47.6%**	**14.21 [-3.20, 31.62]**
Heterogeneity: Tau² = 217.63; Chi² = 11.65, df = 6 (P = 0.07); I² = 48%								
Test for overall effect: Z = 1.60 (P = 0.11)								
**Total (95% CI)**			**333**			**324**	**100.0%**	**19.43 [4.91, 33.94]**
Heterogeneity: Tau² = 404.19; Chi² = 62.96, df = 12 (P < 0.00001); I² = 81%								
Test for overall effect: Z = 2.62 (P = 0.009)								
Test for subgroup differences: Chi² = 0.20, df = 1 (P = 0.66), I² = 0%								

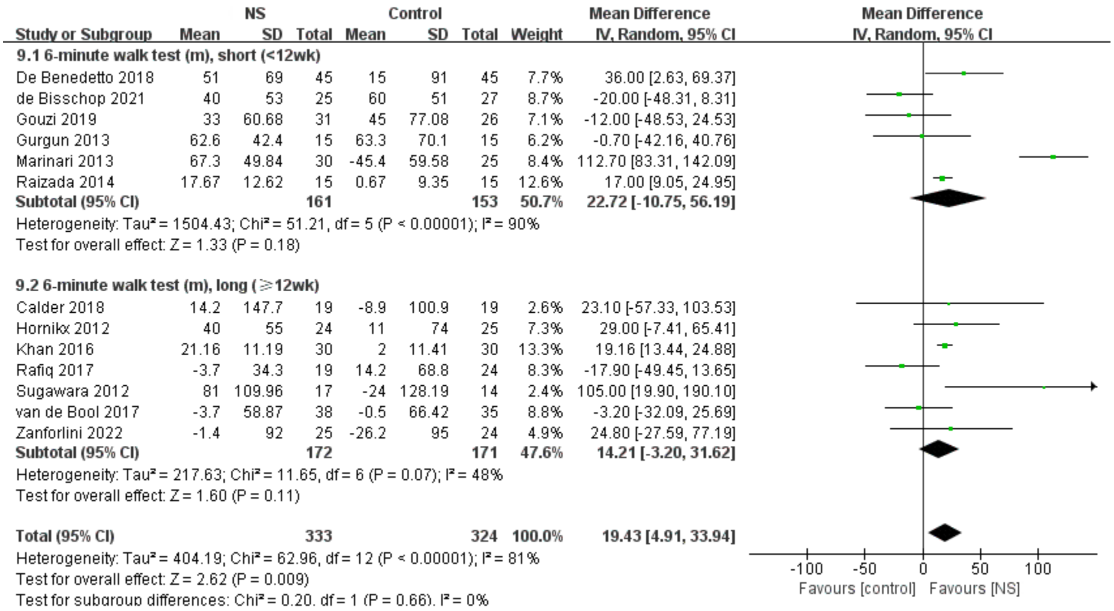

Figure S9. Forest plot of subgroup analysis results from 6-minute walk test changes in response to nutrient supplements based on different intervention periods in people with chronic obstructive pulmonary disease.

Study or Subgroup	NS Mean	NS SD	NS Total	Control Mean	Control SD	Control Total	Weight	Mean Difference IV, Random, 95% CI
**10.1 body weight (kg), presence of pulmonary rehabilitation**								
Aldhahir 2021	1	3.03	22	1	2.94	22	4.9%	0.00 [-1.76, 1.76]
Baldi 2010	3.8	2.6	13	-0.1	1.1	13	5.2%	3.90 [2.37, 5.43]
Broekhuizen 2005	2	2.3	38	1.6	2.7	42	5.9%	0.40 [-0.70, 1.50]
de Bisschop 2021	-0.6	1.6	25	-0.8	1.9	29	6.1%	0.20 [-0.73, 1.13]
Deacon 2008	0.7	1.67	38	0.2	1.93	42	6.3%	0.50 [-0.29, 1.29]
Faager 2006	1.24	3.2	13	1.04	3.1	10	3.6%	0.20 [-2.39, 2.79]
Fuld 2005	0.4	2.9	14	-0.3	1	11	5.1%	0.70 [-0.93, 2.33]
Gurgun 2013	1.1	0.9	15	0.6	0.7	15	6.6%	0.50 [-0.08, 1.08]
Laviolette 2010	-0.09	2.42	10	-1.14	2.59	10	4.2%	1.05 [-1.15, 3.25]
Steiner 2003	0.63	1.5	25	-0.58	1.5	35	6.4%	1.21 [0.44, 1.98]
Sugawara 2012	1.3	1.57	17	-0.2	0.97	14	6.2%	1.50 [0.60, 2.40]
van de Bool 2017	1.9	1.99	38	0.3	2.28	35	6.1%	1.60 [0.61, 2.59]
**Subtotal (95% CI)**			**268**			**278**	**66.5%**	**0.97 [0.48, 1.45]**
Heterogeneity: Tau² = 0.38; Chi² = 26.07, df = 11 (P = 0.006); I² = 58%								
Test for overall effect: Z = 3.88 (P = 0.0001)								
**10.2 body weight (kg), absence of pulmonary rehabilitation**								
Ahmadi 2020	0.52	2.19	23	0.64	2.68	21	5.0%	-0.12 [-1.57, 1.33]
Calder 2018	1.81	2.91	20	1.38	1.74	19	5.0%	0.43 [-1.07, 1.93]
Dal Negro 2012	5.53	3.81	44	-1.89	1.73	44	5.4%	7.42 [6.18, 8.66]
Khan 2016	1.48	1.98	30	-0.17	2.6	30	5.5%	1.65 [0.48, 2.82]
Lewis 1987	1.1	0.63	10	0.3	0.88	11	6.2%	0.80 [0.15, 1.45]
Raizada 2014	1.15	2.02	15	-0.53	2.9	15	4.5%	1.68 [-0.11, 3.47]
**Subtotal (95% CI)**			**142**			**140**	**31.5%**	**1.98 [-0.18, 4.14]**
Heterogeneity: Tau² = 6.82; Chi² = 98.35, df = 5 (P < 0.00001); I² = 95%								
Test for overall effect: Z = 1.80 (P = 0.07)								
**Total (95% CI)**			**410**			**418**	**100.0%**	**1.33 [0.60, 2.05]**
Heterogeneity: Tau² = 1.98; Chi² = 134.18, df = 17 (P < 0.00001); I² = 87%								
Test for overall effect: Z = 3.60 (P = 0.0003)								
Test for subgroup differences: Chi² = 0.81, df = 1 (P = 0.37), I² = 0%								

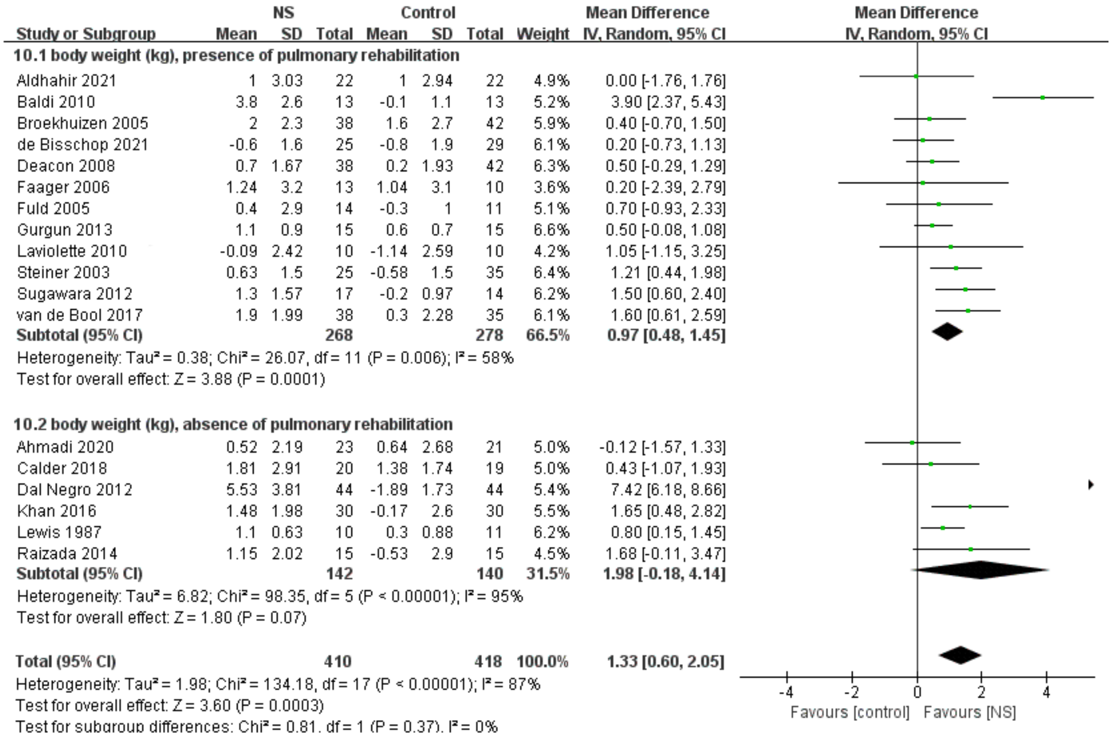

Figure S10. Forest plot of subgroup analysis results from body weight changes in response to nutrient supplements based on the presence or absence of pulmonary rehabilitation in people with chronic obstructive pulmonary disease.

Study or Subgroup	NS Mean	NS SD	NS Total	Control Mean	Control SD	Control Total	Weight	Mean Difference IV, Random, 95% CI
**11.1 body mass index (kg/m²), presence of pulmonary rehabilitation**								
de Bisschop 2021	-0.1	0.7	25	-0.3	1.5	29	7.8%	0.20 [-0.41, 0.81]
Faager 2006	0.4	1.89	13	0.3	0.83	10	5.5%	0.10 [-1.05, 1.25]
Gouzi 2019	-0.3	1.72	31	-0.2	1.26	26	7.1%	-0.10 [-0.88, 0.68]
Gurgun 2013	0.2	1.4	15	0.08	0.2	15	7.4%	0.12 [-0.60, 0.84]
Steiner 2003	0.24	0.5	25	-0.22	0.6	35	9.0%	0.46 [0.18, 0.74]
**Subtotal (95% CI)**			**109**			**115**	**36.8%**	**0.33 [0.11, 0.56]**
Heterogeneity: Tau² = 0.00; Chi² = 2.67, df = 4 (P = 0.61); I² = 0%								
Test for overall effect: Z = 2.89 (P = 0.004)								
**11.2 body mass index (kg/m²), absence of pulmonary rehabilitation**								
Ahmadi 2020	0.17	0.75	23	0.2	0.97	21	8.2%	-0.03 [-0.55, 0.49]
Calder 2018	0.63	1.03	20	0.44	0.57	19	8.2%	0.19 [-0.33, 0.71]
Dal Negro 2012	2.1	1.53	44	-0.31	0.53	44	8.4%	2.41 [1.93, 2.89]
De Benedetto 2018	-0.4	3	45	-0.1	1.2	45	6.4%	-0.30 [-1.24, 0.64]
Khan 2016	0.6	1.41	30	0	1.47	30	7.3%	0.60 [-0.13, 1.33]
Marinari 2013	0.8	3.6	30	-0.4	2.12	25	4.2%	1.20 [-0.33, 2.73]
Pirabbasi 2016	0.09	1.63	13	-0.1	1.27	18	5.9%	0.19 [-0.87, 1.25]
Rafiq 2017	0.01	0.93	19	-0.06	0.96	24	8.0%	0.07 [-0.50, 0.64]
Raizada 2014	0.51	1.32	15	-0.09	1.13	15	6.6%	0.60 [-0.28, 1.48]
**Subtotal (95% CI)**			**239**			**241**	**63.2%**	**0.54 [-0.15, 1.23]**
Heterogeneity: Tau² = 0.94; Chi² = 70.81, df = 8 (P < 0.00001); I² = 89%								
Test for overall effect: Z = 1.55 (P = 0.12)								
**Total (95% CI)**			**348**			**356**	**100.0%**	**0.41 [-0.01, 0.83]**
Heterogeneity: Tau² = 0.49; Chi² = 77.71, df = 13 (P < 0.00001); I² = 83%								
Test for overall effect: Z = 1.92 (P = 0.06)								
Test for subgroup differences: Chi² = 0.33, df = 1 (P = 0.56), I² = 0%								

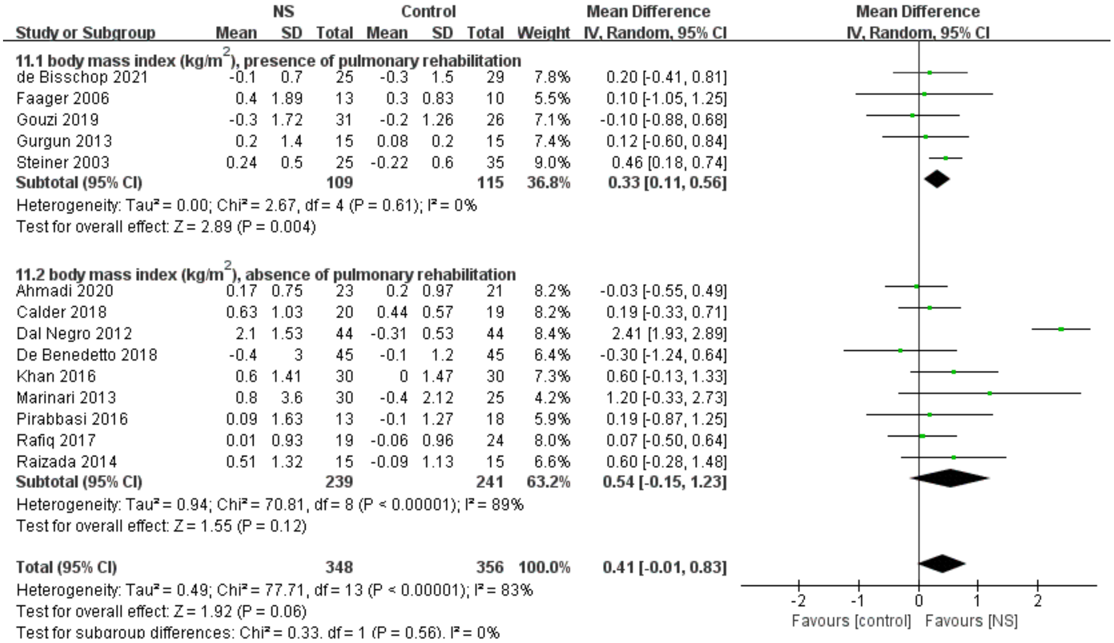

Figure S11. Forest plot of subgroup analysis results from body mass index changes in response to nutrient supplements based on the presence or absence of pulmonary rehabilitation in people with chronic obstructive pulmonary disease.

Study or Subgroup	NS Mean	NS SD	NS Total	Control Mean	Control SD	Control Total	Weight	Mean Difference IV, Random, 95% CI
**12.1 fat-free mass (kg), presence of pulmonary rehabilitation**								
Aldhahir 2021	-3	11.08	22	1	7.16	22	1.4%	-4.00 [-9.51, 1.51]
Baldi 2010	1.5	2.6	13	-0.1	2.3	13	7.9%	1.60 [-0.29, 3.49]
Broekhuizen 2005	1.2	2.2	38	1.1	2.1	42	15.2%	0.10 [-0.84, 1.04]
Deacon 2008	0.9	2.4	38	0.8	2.6	42	13.7%	0.10 [-1.00, 1.20]
Fuld 2005	2	1.6	14	0.4	0.9	11	14.7%	1.60 [0.61, 2.59]
Steiner 2003	0.13	1.37	25	0.63	1.31	35	17.7%	-0.50 [-1.19, 0.19]
Sugawara 2012	0.8	3.59	17	-0.1	1.19	14	8.3%	0.90 [-0.92, 2.72]
**Subtotal (95% CI)**			**167**			**179**	**78.8%**	**0.41 [-0.38, 1.21]**
Heterogeneity: Tau² = 0.63; Chi² = 16.55, df = 6 (P = 0.01); I² = 64%								
Test for overall effect: Z = 1.02 (P = 0.31)								
**12.2 fat-free mass (kg), absence of pulmonary rehabilitation**								
Ahmadi 2020	2.85	4.65	23	0.78	2.62	21	6.4%	2.07 [-0.14, 4.28]
Dal Negro 2012	3.66	2.97	44	3.8	2.95	44	12.4%	-0.14 [-1.38, 1.10]
Pirabbasi 2016	-2.82	6.14	13	-2.07	5.17	18	2.3%	-0.75 [-4.85, 3.35]
**Subtotal (95% CI)**			**80**			**83**	**21.2%**	**0.49 [-1.10, 2.07]**
Heterogeneity: Tau² = 0.78; Chi² = 3.21, df = 2 (P = 0.20); I² = 38%								
Test for overall effect: Z = 0.60 (P = 0.55)								
**Total (95% CI)**			**247**			**262**	**100.0%**	**0.42 [-0.24, 1.08]**
Heterogeneity: Tau² = 0.52; Chi² = 19.77, df = 9 (P = 0.02); I² = 54%								
Test for overall effect: Z = 1.24 (P = 0.21)								
Test for subgroup differences: Chi² = 0.01, df = 1 (P = 0.93), I² = 0%								

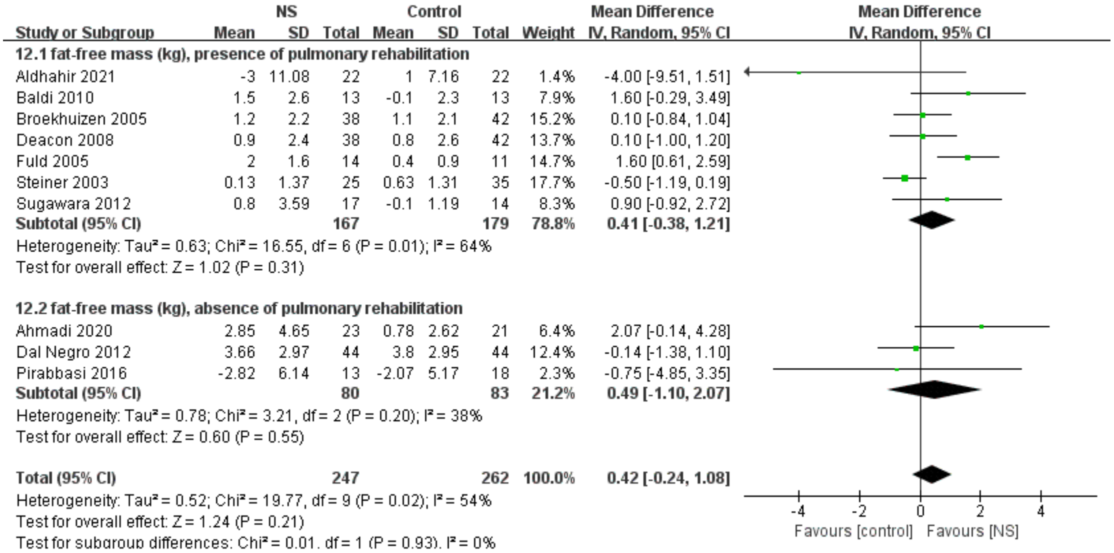

Figure S12. Forest plot of subgroup analysis results from fat-free mass changes in response to nutrient supplements based on the presence or absence of pulmonary rehabilitation in people with chronic obstructive pulmonary disease.

Study or Subgroup	NS Mean	NS SD	NS Total	Control Mean	Control SD	Control Total	Weight	Mean Difference IV, Random, 95% CI
**13.1 fat-free mass index (kg/m^2), presence of pulmonary rehabilitation**								
Aldhahir 2021	1	2.12	22	0	1.91	22	9.7%	1.00 [-0.19, 2.19]
Gouzi 2019	0	1.24	31	0	1.56	26	13.9%	0.00 [-0.74, 0.74]
Gurgun 2013	0.6	0.5	15	0.1	0.6	15	17.2%	0.50 [0.10, 0.90]
Sugawara 2012	0.2	0.78	17	0	0.67	14	16.2%	0.20 [-0.31, 0.71]
**Subtotal (95% CI)**			**85**			**77**	**57.0%**	**0.37 [0.09, 0.65]**
Heterogeneity: Tau² = 0.00; Chi² = 2.87, df = 3 (P = 0.41); I² = 0%								
Test for overall effect: Z = 2.56 (P = 0.01)								
**13.2 fat-free mass index (kg/m^2), absence of pulmonary rehabilitation**								
Ahmadi 2020	0.99	1.61	23	0.29	0.97	21	11.5%	0.70 [-0.08, 1.48]
Dal Negro 2012	1.34	1.52	44	-0.02	1.38	44	13.3%	1.36 [0.75, 1.97]
Marinari 2013	3.7	3.84	30	-0.6	3.31	25	4.2%	4.30 [2.41, 6.19]
Pirabbasi 2016	-1.02	1.87	13	-0.78	1.9	18	6.8%	-0.24 [-1.58, 1.10]
**Subtotal (95% CI)**			**110**			**108**	**35.7%**	**1.33 [0.14, 2.51]**
Heterogeneity: Tau² = 1.11; Chi² = 16.53, df = 3 (P = 0.0009); I² = 82%								
Test for overall effect: Z = 2.20 (P = 0.03)								
**Total (95% CI)**			**195**			**185**	**100.0%**	**0.74 [0.21, 1.27]**
Heterogeneity: Tau² = 0.39; Chi² = 27.86, df = 7 (P = 0.0002); I² = 75%								
Test for overall effect: Z = 2.72 (P = 0.007)								
Test for subgroup differences: Chi² = 2.40, df = 1 (P = 0.12), I² = 58.3%								

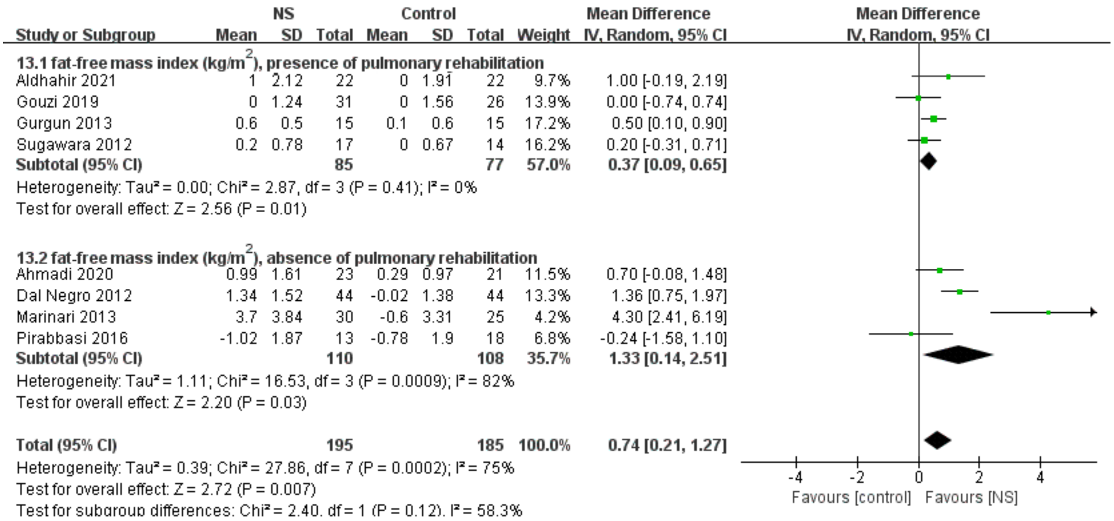

Figure S13. Forest plot of subgroup analysis results from fat-free mass index changes in response to nutrient supplements based on the presence or absence of pulmonary rehabilitation in people with chronic obstructive pulmonary disease.

Study or Subgroup	NS Mean	NS SD	NS Total	Control Mean	Control SD	Control Total	Weight	Std. Mean Difference IV, Random, 95% CI
**14.1 handgrip strength, presence of pulmonary rehabilitation**								
Aldhahir 2021	2	5.5	22	3	12.16	22	10.1%	-0.10 [-0.70, 0.49]
Faager 2006	6.8	39.18	12	16.6	60.37	10	8.6%	-0.19 [-1.03, 0.65]
Steiner 2003	0.64	1.6	25	-0.05	2.1	35	10.5%	0.36 [-0.16, 0.87]
**Subtotal (95% CI)**			**59**			**67**	**29.3%**	**0.10 [-0.26, 0.45]**
Heterogeneity: Tau² = 0.00; Chi² = 1.86, df = 2 (P = 0.40); I² = 0%								
Test for overall effect: Z = 0.53 (P = 0.59)								
**14.2 handgrip strength, absence of pulmonary rehabilitation**								
Ahmadi 2020	2.76	3.81	23	0.2	4.54	21	10.0%	0.60 [-0.00, 1.21]
Dal Negro 2012	1.6	0.85	44	-0.6	0.97	44	10.3%	2.39 [1.84, 2.94]
De Benedetto 2018	0.71	4.18	20	-0.14	3.26	20	9.9%	0.22 [-0.40, 0.84]
Deutz 2021	1.56	7	109	-0.34	6.5	105	11.6%	0.28 [0.01, 0.55]
Lewis 1987	1.8	7.17	10	0.5	13.47	11	8.6%	0.11 [-0.74, 0.97]
Rafiq 2017	-0.04	2.73	19	0.16	3.89	24	10.0%	-0.06 [-0.66, 0.54]
Zanforlini 2022	-0.1	4	25	1	4.9	24	10.3%	-0.24 [-0.80, 0.32]
**Subtotal (95% CI)**			**250**			**249**	**70.7%**	**0.48 [-0.15, 1.11]**
Heterogeneity: Tau² = 0.63; Chi² = 58.85, df = 6 (P < 0.00001); I² = 90%								
Test for overall effect: Z = 1.49 (P = 0.14)								
**Total (95% CI)**			**309**			**316**	**100.0%**	**0.35 [-0.11, 0.81]**
Heterogeneity: Tau² = 0.46; Chi² = 63.62, df = 9 (P < 0.00001); I² = 86%								
Test for overall effect: Z = 1.49 (P = 0.14)								
Test for subgroup differences: Chi² = 1.09, df = 1 (P = 0.30), I² = 7.8%								

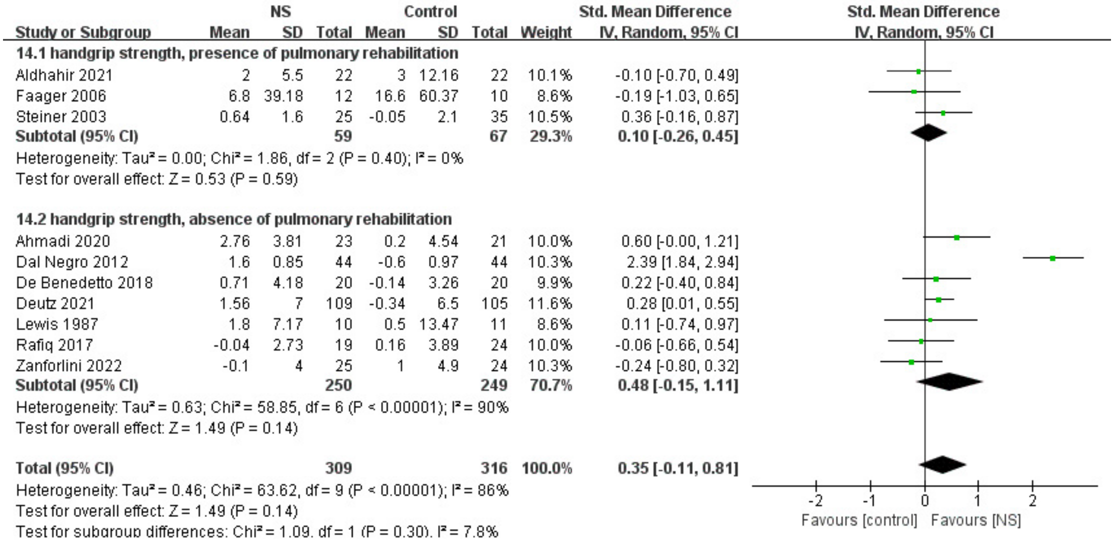

Figure S14. Forest plot of subgroup analysis results from handgrip strength changes in response to nutrient supplements based on the presence or absence of pulmonary rehabilitation in people with chronic obstructive pulmonary disease.

Study or Subgroup	NS Mean	NS SD	NS Total	Control Mean	Control SD	Control Total	Weight	Std. Mean Difference IV, Fixed, 95% CI
**15.1 quadriceps muscle strength, presence of pulmonary rehabilitation**								
Ahnfeldt-Mollerup 2015	0.27	0.7	28	0.22	0.63	25	9.2%	0.07 [-0.47, 0.61]
Broekhuizen 2005	-2	27.91	7	21	29.44	5	1.8%	-0.74 [-1.95, 0.46]
de Bisschop 2021	3.2	3.8	24	4.1	5.3	29	9.1%	-0.19 [-0.73, 0.35]
Faager 2006	19.6	11.1	38	23.1	17	42	13.8%	-0.24 [-0.68, 0.20]
Fuld 2005	9	21	38	12	24	42	13.9%	-0.13 [-0.57, 0.31]
Gouzi 2019	9	26.03	31	0	35.73	26	9.7%	0.29 [-0.24, 0.81]
Hornikx 2012	15	16	24	7	19	25	8.3%	0.45 [-0.12, 1.02]
Laviolette 2010	2.6	6	10	-3	8.24	10	3.2%	0.74 [-0.17, 1.66]
Steiner 2003	17.4	34.28	25	3.6	34.06	35	10.0%	0.40 [-0.12, 0.92]
van de Bool 2017	13.6	31.86	38	10.8	29.27	35	12.7%	0.09 [-0.37, 0.55]
**Subtotal (95% CI)**			**263**			**274**	**91.7%**	**0.07 [-0.10, 0.24]**
Heterogeneity: Chi² = 11.34, df = 9 (P = 0.25); I² = 21%								
Test for overall effect: Z = 0.81 (P = 0.42)								
**15.2 quadriceps muscle strength, absence of pulmonary rehabilitation**								
Zanforlini 2022	5.1	20.5	25	-5.6	22	24	7.9%	0.50 [-0.07, 1.06]
**Subtotal (95% CI)**			**25**			**24**	**7.9%**	**0.50 [-0.07, 1.06]**
Heterogeneity: Not applicable								
Test for overall effect: Z = 1.71 (P = 0.09)								
**Total (95% CI)**			**288**			**298**	**100.0%**	**0.11 [-0.06, 0.27]**
Heterogeneity: Chi² = 13.30, df = 10 (P = 0.21); I² = 25%								
Test for overall effect: Z = 1.27 (P = 0.20)								
Test for subgroup differences: Chi² = 1.96, df = 1 (P = 0.16), I² = 49.0%								

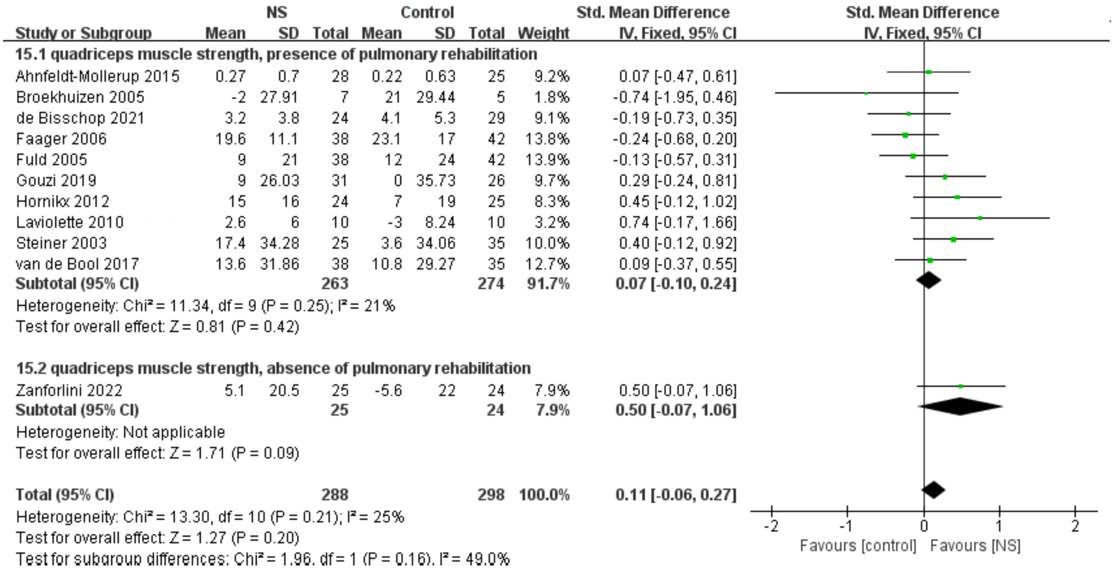

Figure S15. Forest plot of subgroup analysis results from quadriceps muscle strength changes in response to nutrient supplements based on the presence or absence of pulmonary rehabilitation in people with chronic obstructive pulmonary disease.

Study or Subgroup	NS Mean	NS SD	NS Total	Control Mean	Control SD	Control Total	Weight	Mean Difference IV, Random, 95% CI
**16.1 6-minute walk test (m), presence of pulmonary rehabilitation**								
de Bisschop 2021	40	53	25	60	51	27	9.0%	-20.00 [-48.31, 8.31]
Gouzi 2019	33	60.68	31	45	77.08	26	7.3%	-12.00 [-48.53, 24.53]
Gurgun 2013	62.6	42.4	15	63.3	70.1	15	6.4%	-0.70 [-42.16, 40.76]
Hornikx 2012	40	55	24	11	74	25	7.3%	29.00 [-7.41, 65.41]
Sugawara 2012	81	109.96	17	-24	128.19	14	2.4%	105.00 [19.90, 190.10]
van de Bool 2017	-3.7	58.87	38	-0.5	66.42	35	8.8%	-3.20 [-32.09, 25.69]
**Subtotal (95% CI)**			**150**			**142**	**41.2%**	**3.94 [-18.56, 26.44]**
Heterogeneity: Tau² = 397.34; Chi² = 10.66, df = 5 (P = 0.06); I² = 53%								
Test for overall effect: Z = 0.34 (P = 0.73)								
**16.2 6-minute walk test (m), absence of pulmonary rehabilitation**								
Calder 2018	14.2	147.7	19	-8.9	100.9	19	2.5%	23.10 [-57.33, 103.53]
De Benedetto 2018	51	69	45	15	91	45	7.7%	36.00 [2.63, 69.37]
Khan 2016	21.16	11.19	30	2	11.41	30	12.8%	19.16 [13.44, 24.88]
Marinari 2013	67.3	49.84	30	-45.4	59.58	25	8.4%	112.70 [83.31, 142.09]
Rafiq 2017	-3.7	34.3	19	14.2	68.8	24	8.0%	-17.90 [-49.45, 13.65]
Raizada 2014	17.67	12.62	15	0.67	9.35	15	12.6%	17.00 [9.05, 24.95]
Zanforlini 2022	-1.4	92	25	-26.2	95	24	4.8%	24.80 [-27.59, 77.19]
**Subtotal (95% CI)**			**183**			**182**	**56.9%**	**30.21 [11.51, 48.92]**
Heterogeneity: Tau² = 390.54; Chi² = 45.31, df = 6 (P < 0.00001); I² = 87%								
Test for overall effect: Z = 3.17 (P = 0.002)								
**Total (95% CI)**			**333**			**324**	**100.0%**	**19.43 [4.91, 33.94]**
Heterogeneity: Tau² = 404.19; Chi² = 62.96, df = 12 (P < 0.00001); I² = 81%								
Test for overall effect: Z = 2.62 (P = 0.009)								
Test for subgroup differences: Chi² = 3.10, df = 1 (P = 0.08), I² = 67.7%								

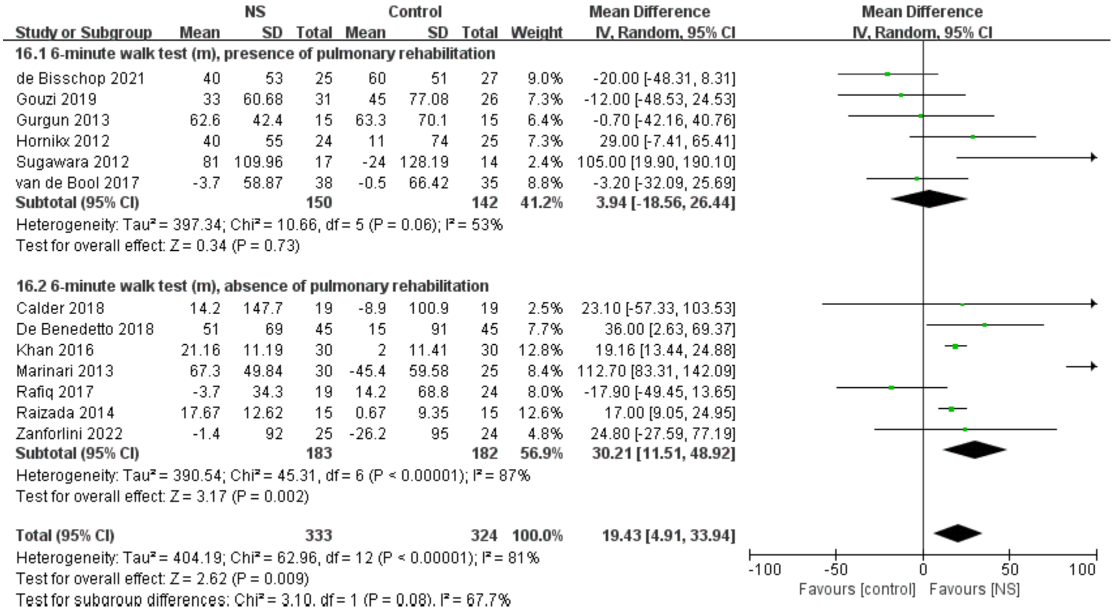

Figure S16. Forest plot of subgroup analysis results from 6-minute walk test changes in response to nutrient supplements based on the presence or absence of pulmonary rehabilitation in people with chronic obstructive pulmonary disease.

Study or Subgroup	NS Mean	NS SD	NS Total	Control Mean	Control SD	Control Total	Weight	Mean Difference IV, Fixed, 95% CI
**17.1 body mass index (kg/m^2), energy nutrient supplements**								
Raizada 2014	0.51	1.32	15	-0.09	1.13	15	6.5%	0.60 [-0.28, 1.48]
Steiner 2003	0.24	0.5	25	-0.22	0.6	35	64.9%	0.46 [0.18, 0.74]
**Subtotal (95% CI)**			**40**			**50**	**71.4%**	**0.47 [0.21, 0.74]**
Heterogeneity: Chi² = 0.09, df = 1 (P = 0.77); I² = 0%								
Test for overall effect: Z = 3.48 (P = 0.0005)								
**17.2 body mass index (kg/m^2), non-energy nutrient supplements**								
Gouzi 2019	-0.3	1.72	31	-0.2	1.26	26	8.4%	-0.10 [-0.88, 0.68]
Pirabbasi 2016	0.09	1.63	13	-0.1	1.27	18	4.5%	0.19 [-0.87, 1.25]
Rafiq 2017	0.01	0.93	19	-0.06	0.96	24	15.7%	0.07 [-0.50, 0.64]
**Subtotal (95% CI)**			**63**			**68**	**28.6%**	**0.04 [-0.38, 0.46]**
Heterogeneity: Chi² = 0.21, df = 2 (P = 0.90); I² = 0%								
Test for overall effect: Z = 0.18 (P = 0.86)								
**Total (95% CI)**			**103**			**118**	**100.0%**	**0.35 [0.12, 0.57]**
Heterogeneity: Chi² = 3.22, df = 4 (P = 0.52); I² = 0%								
Test for overall effect: Z = 3.04 (P = 0.002)								
Test for subgroup differences: Chi² = 2.92, df = 1 (P = 0.09), I² = 65.8%								

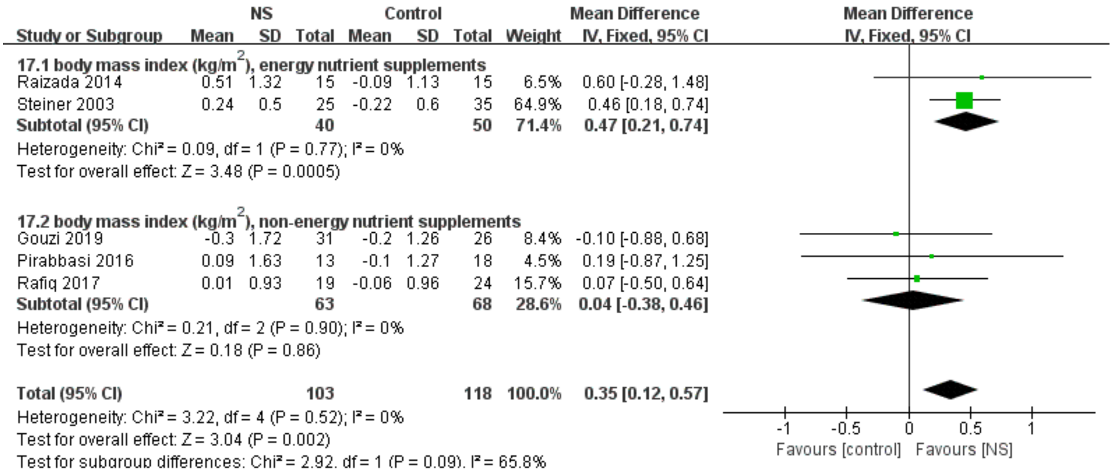

Figure S17. Forest plot of subgroup analysis results from body mass index changes in response to nutrient supplements (NS) based on the types of NS in people with chronic obstructive pulmonary disease.

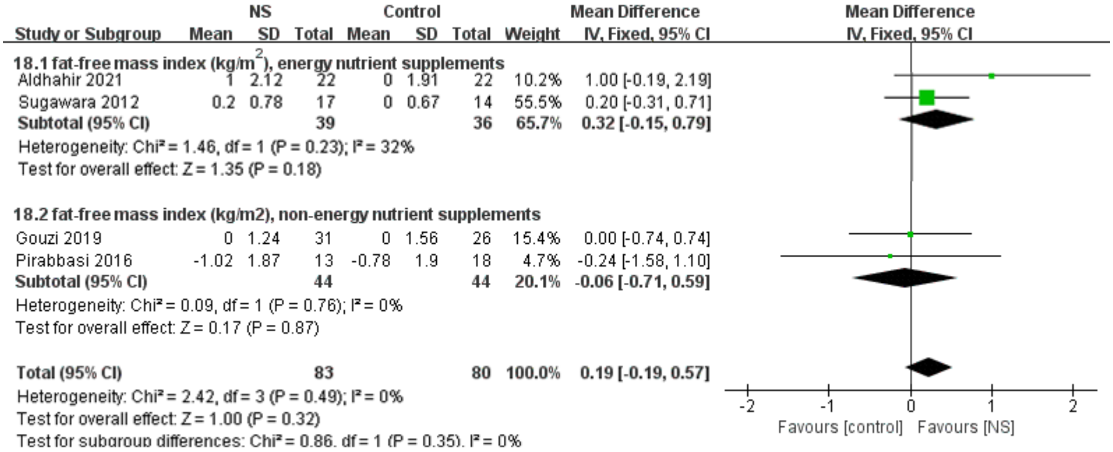

Figure S18. Forest plot of subgroup analysis results from fat-free mass index changes in response to nutrient supplements (NS) based on the types of NS in people with chronic obstructive pulmonary disease.

	NS			Control				Std. Mean Difference
Study or Subgroup	Mean	SD	Total	Mean	SD	Total	Weight	IV, Fixed, 95% CI
**19.1 handgrip strength, energy nutrient supplements**								
Aldhahir 2021	2	5.5	22	3	12.16	22	10.3%	-0.10 [-0.70, 0.49]
Deutz 2021	1.56	7	109	-0.34	6.5	105	49.8%	0.28 [0.01, 0.55]
Lewis 1987	1.8	7.17	10	0.5	13.47	11	4.9%	0.11 [-0.74, 0.97]
Steiner 2003	0.64	1.6	25	-0.05	2.1	35	13.5%	0.36 [-0.16, 0.87]
**Subtotal (95% CI)**			**166**			**173**	**78.6%**	**0.23 [0.02, 0.45]**
Heterogeneity: Chi² = 1.66, df = 3 (P = 0.65); I² = 0%								
Test for overall effect: Z = 2.12 (P = 0.03)								
**19.2 handgrip strength, non-energy nutrient supplements**								
Rafiq 2017	-0.04	2.73	19	0.16	3.89	24	10.0%	-0.06 [-0.66, 0.54]
Zanforlini 2022	-0.1	4	25	1	4.9	24	11.4%	-0.24 [-0.80, 0.32]
**Subtotal (95% CI)**			**44**			**48**	**21.4%**	**-0.16 [-0.57, 0.25]**
Heterogeneity: Chi² = 0.19, df = 1 (P = 0.66); I² = 0%								
Test for overall effect: Z = 0.74 (P = 0.46)								
**Total (95% CI)**			**210**			**221**	**100.0%**	**0.15 [-0.04, 0.34]**
Heterogeneity: Chi² = 4.55, df = 5 (P = 0.47); I² = 0%								
Test for overall effect: Z = 1.54 (P = 0.12)								
Test for subgroup differences: Chi² = 2.70, df = 1 (P = 0.10), I² = 62.9%								

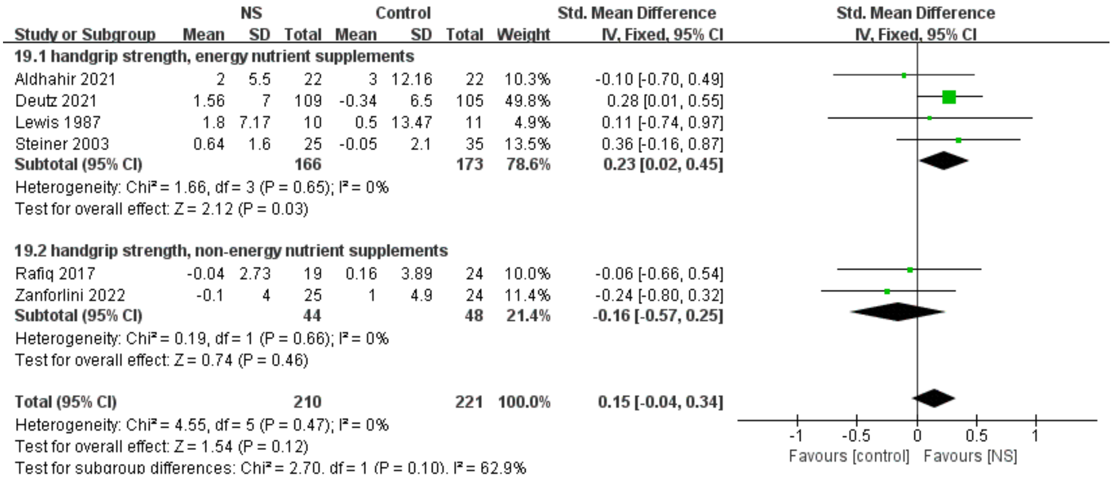

Figure S19. Forest plot of subgroup analysis results from handgrip strength changes in response to nutrient supplements (NS) based on the types of NS in people with chronic obstructive pulmonary disease.

Study or Subgroup	NS Mean	NS SD	NS Total	Control Mean	Control SD	Control Total	Weight	Std. Mean Difference IV, Fixed, 95% CI
**20.1 quadriceps muscle strength, energy nutrient supplements**								
Steiner 2003	17.4	34.28	25	3.6	34.06	35	20.3%	0.40 [-0.12, 0.92]
van de Bool 2017	13.6	31.86	38	10.8	29.27	35	25.9%	0.09 [-0.37, 0.55]
**Subtotal (95% CI)**			**63**			**70**	**46.2%**	**0.23 [-0.12, 0.57]**
Heterogeneity: Chi² = 0.76, df = 1 (P = 0.38); I² = 0%								
Test for overall effect: Z = 1.29 (P = 0.20)								
**20.2 quadriceps muscle strength, non-energy nutrient supplements**								
Gouzi 2019	9	26.03	31	0	35.73	26	18.0%	0.29 [-0.24, 0.81]
Hornikx 2012	15	16	24	7	19	25	15.4%	0.45 [-0.12, 1.02]
Zanforlini 2022	5.1	20.5	25	-5.6	22	24	15.3%	0.50 [-0.07, 1.06]
**Subtotal (95% CI)**			**80**			**75**	**48.7%**	**0.40 [0.08, 0.72]**
Heterogeneity: Chi² = 0.31, df = 2 (P = 0.86); I² = 0%								
Test for overall effect: Z = 2.48 (P = 0.01)								
**Total (95% CI)**			**143**			**145**	**100.0%**	**0.32 [0.09, 0.56]**
Heterogeneity: Chi² = 1.62, df = 4 (P = 0.81); I² = 0%								
Test for overall effect: Z = 2.69 (P = 0.007)								
Test for subgroup differences: Chi² = 0.55, df = 1 (P = 0.46), I² = 0%								

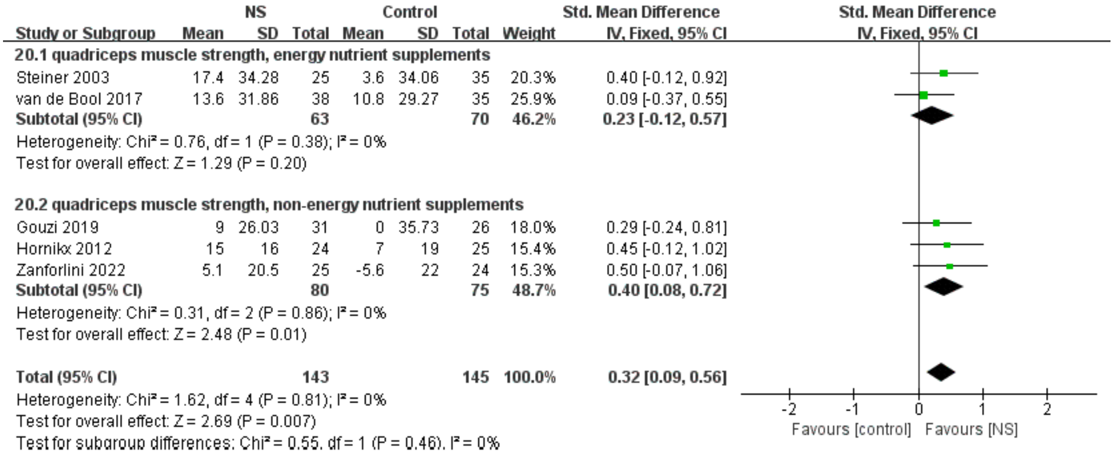

Figure S20. Forest plot of subgroup analysis results from quadriceps muscle strength changes in response to nutrient supplements (NS) based on the types of NS in people with chronic obstructive pulmonary disease.

Study or Subgroup	NS Mean	NS SD	NS Total	Control Mean	Control SD	Control Total	Weight	Mean Difference IV, Random, 95% CI
**21.1 6-minute walk test (m), energy nutrient supplements**								
Raizada 2014	17.67	12.62	15	0.67	9.35	15	29.6%	17.00 [9.05, 24.95]
Sugawara 2012	81	109.96	17	-24	128.19	14	3.7%	105.00 [19.90, 190.10]
van de Bool 2017	-3.7	58.87	38	-0.5	66.42	35	16.8%	-3.20 [-32.09, 25.69]
**Subtotal (95% CI)**			**70**			**64**	**50.1%**	**18.45 [-11.30, 48.20]**
Heterogeneity: Tau² = 414.38; Chi² = 5.95, df = 2 (P = 0.05); I² = 66%								
Test for overall effect: Z = 1.22 (P = 0.22)								
**21.2 6-minute walk test (m), non-energy nutrient supplements**								
Gouzi 2019	33	60.68	31	45	77.08	26	12.7%	-12.00 [-48.53, 24.53]
Hornikx 2012	40	55	24	11	74	25	12.7%	29.00 [-7.41, 65.41]
Rafiq 2017	-3.7	34.3	19	14.2	68.8	24	14.8%	-17.90 [-49.45, 13.65]
Zanforlini 2022	-1.4	92	25	-26.2	95	24	7.9%	24.80 [-27.59, 77.19]
**Subtotal (95% CI)**			**99**			**99**	**48.1%**	**2.98 [-21.42, 27.39]**
Heterogeneity: Tau² = 241.12; Chi² = 4.93, df = 3 (P = 0.18); I² = 39%								
Test for overall effect: Z = 0.24 (P = 0.81)								
**Total (95% CI)**			**169**			**163**	**100.0%**	**9.85 [-7.48, 27.18]**
Heterogeneity: Tau² = 247.59; Chi² = 12.99, df = 6 (P = 0.04); I² = 54%								
Test for overall effect: Z = 1.11 (P = 0.27)								
Test for subgroup differences: Chi² = 0.62, df = 1 (P = 0.43), I² = 0%								

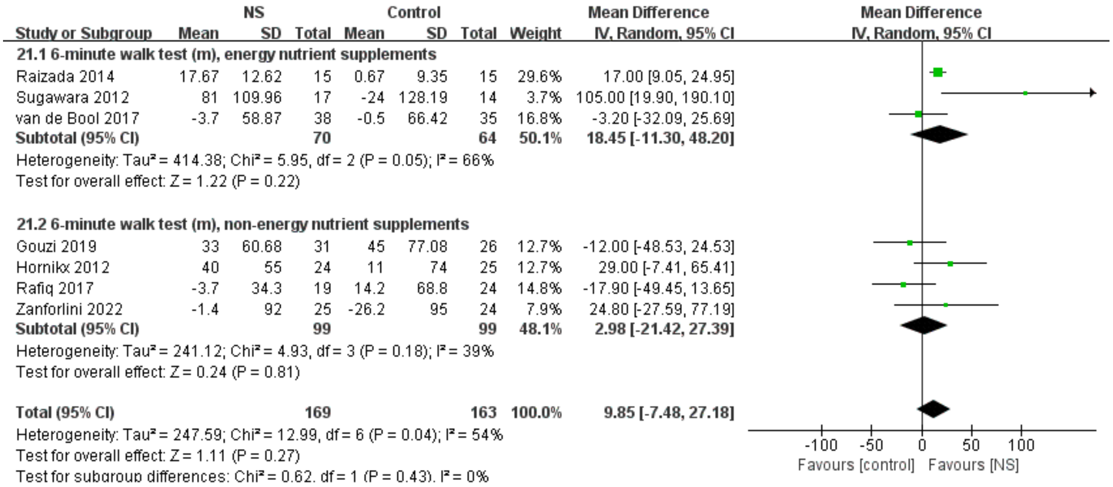

Figure S21. Forest plot of subgroup analysis results from 6-minute walk test changes in response to nutrient supplements (NS) based on the types of NS in people with chronic obstructive pulmonary disease.

Table S1. Search formulas employed to screen electronic databases for literature search

PubMed	Search for query	Results
#1	"Pulmonary Disease, Chronic Obstructive"[Mesh]	64,282
#2	((((((((((Chronic Obstructive Lung Disease[Title/Abstract]) OR (Chronic Obstructive Pulmonary Diseases[Title/Abstract])) OR (COAD[Title/Abstract])) OR (COPD[Title/Abstract])) OR (Chronic Obstructive Airway Disease[Title/Abstract])) OR (Chronic Obstructive Pulmonary Disease[Title/Abstract])) OR (Airflow Obstruction, Chronic[Title/Abstract])) OR (Airflow Obstructions, Chronic[Title/Abstract])) OR (Chronic Airflow Obstructions[Title/Abstract])) OR (Chronic Airflow Obstruction[Title/Abstract]))	79,298
#3	#1 OR #2	100,554
#4	((((((((((((((supplement*) OR (nutritional intervention*)) OR (nutrition intervention*)) OR (nutrition support)) OR (nutritional support)) OR (nutritional recommendation)) OR (nutrition recommendation)) OR (nutritional therapy)) OR (nutrition therapy)) OR (enriched meal*)) OR (ONS)) OR (FSMP)) OR (sip feed)) OR (sip nutrition)) OR (oral nutrition)	841,984
#5	"Sarcopenia"[Mesh]	7,913
#6	((sarcopeni*[Title/Abstract])) OR (myopeni*[Title/Abstract])) OR (muscle loss[Title/Abstract])) OR (muscle depletion[Title/Abstract])) OR (muscle wasting[Title/Abstract])) OR (muscle reduction[Title/Abstract])) OR (reduced muscle[Title/Abstract])) OR (depleted muscle[Title/Abstract])) OR (muscle attenuation[Title/Abstract])) OR (muscle alteration[Title/Abstract])) OR (loss of muscle[Title/Abstract])) OR ("Muscular Atrophy"[Mesh])) OR (muscular atrophy[Title/Abstract])) OR (muscle atrophy[Title/Abstract])) OR ("Muscle, Skeletal"[Mesh])) OR (skeletal muscle[Title/Abstract])) OR (muscle mass[Title/Abstract])) OR (psoas muscle[Title/Abstract])) OR (fat free mass[Title/Abstract])) OR (lean mass[Title/Abstract])) OR (muscle index[Title/Abstract])) OR (muscle size[Title/Abstract])) OR (muscle thickness[Title/Abstract])) OR ("Body Composition"[Mesh])) OR (body composition[Title/Abstract])) OR ("Malnutrition"[Mesh])) OR (malnutrition[Title/Abstract])) OR ("Muscle Strength"[Mesh])) OR (muscle strength[Title/Abstract])) OR (muscular strength[Title/Abstract])) OR (muscle power[Title/Abstract])) OR (muscle function[Title/Abstract])) OR ("Hand Strength"[Mesh])) OR (Hand Strength[Title/Abstract])) OR (handgrip strength[Title/Abstract])) OR (grip	677,897

	strength[Title/Abstract])) OR ("Muscle Fatigue"[Mesh])) OR (muscle fatigue[Title/Abstract])) OR ("Muscle Weakness"[Mesh])) OR (muscle weakness[Title/Abstract])) OR (muscular weakness[Title/Abstract])	
#7	#5 OR #6	677,897
#8	"randomized controlled trial[Publication Type] OR randomized[Title/Abstract] OR placebo[Title/Abstract] "	962,772
#9	#3 AND #4 AND #7 AND #8	143
Embase		
#1	'chronic obstructive lung disease'/exp	160398
#2	chronic obstructive lung disease:ab,ti OR chronic obstructive pulmonary diseases:ab,ti OR coad:ab,ti OR copd:ab,ti OR chronic obstructive airway disease:ab,ti OR chronic obstructive pulmonary disease:ab,ti OR airflow obstruction, chronic:ab,ti OR airflow obstructions, chronic:ab, ti OR chronic airflow obstructions:ab,ti OR chronic airflow obstruction:ab,ti	133746
#3	supplement* OR (nutritional AND intervention*) OR (nutrition AND intervention*) OR (nutrition AND support) OR (nutritional AND support) OR (nutritional AND recommendation) OR (nutrition AND recommendation) OR (nutritional AND therapy) OR (nutrition AND therapy) OR (enriched AND meal*) OR ons OR fsmp OR (sip AND feed) OR (sip AND nutrition) OR (oral AND nutrition)	2610573
#4	sarcopenia/exp	16356
#5	muscle atrophy/exp	53789
#6	skeletal muscle/exp	403428
#7	body composition/exp	118449
#8	malnutrition/exp	193323
#9	muscle strength/exp	83882
#10	hand strength/exp	34844
#11	muscle fatigue/exp	14083
#12	muscle weakness/exp	423971
#13	sarcopeni*:ab,ti OR myopeni*:ab,ti OR muscle loss:ab,ti OR muscle depletion:ab,ti OR muscle wasting:ab,ti OR muscle reduction:ab,ti OR reduced muscle:ab,ti OR depleted muscle:ab,ti OR muscle attenuation:ab,ti OR muscle	280184

	alteration:ab,ti OR loss of muscle:ab,ti OR muscular atrophy:ab,ti OR muscle, skeletal:ab,ti OR muscle mass:ab,ti OR psoas muscle:ab,ti OR fat free mass:ab,ti OR lean mass:ab,ti OR muscle index:ab,ti OR muscle size:ab,ti OR muscle thick ness:ab,ti OR body composition:ab,ti OR malnutrition:ab,ti OR muscle strength:ab,ti OR muscular strength:ab,ti OR muscle power:ab,ti OR muscle function:ab,ti OR hand strength:ab,ti OR handgrip strength:ab,ti OR grip strength:ab,ti OR mu scle fatigue:ab,ti OR muscle weakness:ab,ti OR muscular weakness:ab,ti	
#14	randomized controlled trial:ab,ti OR randomized:ab,ti OR placebo:ab,ti	1051911
#15	#1 OR #2	186603
#16	#4 OR #5 OR #6 OR #7 OR #8 OR #9 OR #10 OR #11 OR #12 OR #13	1223202
#17	#3 AND #14 AND #15 AND #16	243
The Cochrane Library		
#1	MeSH descriptor: [Pulmonary Disease, Chronic Obstructive] explode all trees	6337
#2	(Chronic Obstructive Lung Disease):ti,ab,kw OR (Chronic Obstructive Pulmonary Diseases):ti,ab,kw OR (COAD):ti,ab,kw OR (COPD):ti,ab,kw OR (Chronic Obstructive Airway Disease):ti,ab,kw OR (Chronic Obstructive Pulmonary Disease):ti,ab,kw OR (Airflow Obstruction, Chronic):ti,ab,kw OR (Airflow Obstructions, Chronic):ti,ab,kw OR (Chronic Airflow Obstructions):ti,ab,kw OR (Chronic Airflow Obstruction):ti,ab,kw	22954
#3	#1 or #2	23242
#4	(supplement*) OR (nutritional intervention*) OR (nutrition intervention*) OR (nutrition support) OR (nutritional support) OR (nutritional recommendation) OR (nutrition recommendation) OR (nutritional therapy) OR (nutrition therapy) OR (enriched meal*) OR (ONS) OR (FSMP) OR (sip feed) OR (sip nutrition) OR (oral nutrition)	139294
#5	MeSH descriptor: [Sarcopenia] explode all trees	616
#6	MeSH descriptor: [Muscular Atrophy] explode all trees	990
#7	MeSH descriptor: [Muscle, Skeletal] explode all trees	13303
#8	MeSH descriptor: [Body Composition] explode all trees	5697
#9	MeSH descriptor: [Malnutrition] explode all trees	4728
#10	MeSH descriptor: [Muscle Strength] explode all trees	6700
#11	MeSH descriptor: [Hand Strength] explode all trees	1725

#12	MeSH descriptor: [Muscle Fatigue] explode all trees	1050
#13	MeSH descriptor: [Muscle Weakness] explode all trees	609
#14	(sarcopeni*):ti,ab,kw OR (myopeni*):ti,ab,kw OR (muscle loss):ti,ab,kw OR (muscle depletion):ti,ab,kw OR (muscle wasting):ti,ab,kw OR (muscle reduction):ti,ab,kw OR (reduced muscle):ti,ab,kw OR (depleted muscle):ti,ab,kw OR (muscle attenuation):ti,ab,kw OR (muscle alteration):ti,ab,kw OR (loss of muscle):ti,ab,kw OR (muscular atrophy):ti,ab,kw OR (muscle atrophy):ti,ab,kw OR (skeletal muscle):ti,ab,kw OR (muscle mass):ti,ab,kw OR (psoas muscle):ti,ab,kw OR (fat free mass):ti,ab,kw OR (lean mass):ti,ab,kw OR (muscle index):ti,ab,kw OR (muscle size):ti,ab,kw OR (muscle thickness):ti,ab,kw OR (body composition):ti,ab,kw OR (malnutrition):ti,ab,kw OR (muscle strength):ti,ab,kw OR (muscular strength):ti,ab,kw OR (muscle power):ti,ab,kw OR (muscle function):ti,ab,kw OR (Hand Strength):ti,ab,kw OR (handgrip strength):ti,ab,kw OR (grip strength):ti,ab,kw OR (muscle fatigue):ti,ab,kw OR (muscle weakness):ti,ab,kw OR (muscular weakness):ti,ab,kw	86904
#15	#5 or #6 or #7 or #8 or #9 or #10 or #11 or #12 or #13 or #14	92503
#16	#3 AND #4 AND #15	371
Web of Science		
#1	TS=(Pulmonary Disease, Chronic Obstructive or Chronic Obstructive Lung Disease or Chronic Obstructive Pulmonary Diseases or COAD or COPD or Chronic Obstructive Airway Disease or Chronic Obstructive Pulmonary Disease or Airflow Obstruction, Chronic or Airflow Obstructions, Chronic or Chronic Airflow Obstructions or Chronic Airflow Obstruction)	105,404
#2	TS=(supplement* or nutritional intervention* or nutrition intervention* or nutrition support or nutritional support or nutritional recommendation or nutrition recommendation or nutritional therapy or nutrition therapy or enriched meal* or ONS or FSMP or sip feed or sip nutrition or oral nutrition)	694,069
#3	TS=(Sarcopenia or sarcopeni* or myopeni* or muscle loss or muscle depletion or muscle wasting or muscle reduction or reduced muscle or depleted muscle or muscle attenuation or muscle alteration or loss of muscle or Muscular Atrophy or muscular atrophy or muscle atrophy or Muscle, Skeletal or skeletal muscle or muscle mass or psoas muscle or fat free mass or lean mass or muscle index or muscle size or muscle thickness or Body Composition or body composition or Malnutrition or malnutrition or Muscle Strength or muscle strength or muscular strength or muscle power or muscle	859,315

	function or Hand Strength or Hand Strength or handgrip strength or grip strength or Muscle Fatigue or muscle fatigue or Muscle Weakness or muscle weakness or muscular weakness)	
#4	TS=(randomized controlled trial or randomized or placebo)	1,117,630
#5	#1 AND #2 AND #3 AND #4	187
Ovid		
#1	(Pulmonary Disease, Chronic Obstructive or Chronic Obstructive Lung Disease or Chronic Obstructive Pulmonary Diseases or COAD or COPD or Chronic Obstructive Airway Disease or Chronic Obstructive Pulmonary Disease or Airflow Obstruction, Chronic or Airflow Obstructions, Chronic or Chronic Airflow Obstructions or Chronic Airflow Obstruction).ti,ab,kw.	78236
#2	(supplement* or nutritional intervention* or nutrition intervention* or nutrition support or nutritional support or nutritional recommendation or nutrition recommendation or nutritional therapy or nutrition therapy or enriched meal* or ONS or FSMP or sip feed or sip nutrition or oral nutrition).ti,ab,kw.	410475
#3	(Sarcopenia or sarcopeni* or myopeni* or muscle loss or muscle depletion or muscle wasting or muscle reduction or reduced muscle or depleted muscle or muscle attenuation or muscle alteration or loss of muscle or Muscular Atrophy or muscular atrophy or muscle atrophy or Muscle, Skeletal or skeletal muscle or muscle mass or psoas muscle or fat free mass or lean mass or muscle index or muscle size or muscle thickness or Body Composition or body composition or Malnutrition or malnutrition or Muscle Strength or muscle strength or muscular strength or muscle power or muscle function or Hand Strength or Hand Strength or handgrip strength or grip strength or Muscle Fatigue or muscle fatigue or Muscle Weakness or muscle weakness or muscular weakness).ti,ab,kw.	302186
#4	(randomized controlled trial or randomized or placebo).ti,ab,kw.	737776
#5	1 AND 2 AND 3 AND 4	76
Scopus		

#1	TITLE-ABS-KEY ("pulmonary disease, chronic obstructive" OR "chronic obstructive lung disease" OR "chronic obstructive pulmonary diseases" OR "coad" OR "copd" OR "chronic obstructive airway disease" OR "chronic obstructive pulmonary disease" OR "airflow obstruction, chronic" OR "airflow obstructions, chronic" OR "chronic airflow obstructions" OR "chronic airflow obstruction")<br>Show less	145,607
#2	ALL ("supplement*" OR "nutritional intervention*" OR "nutrition intervention*" OR "nutrition support" OR "nutritional support" OR "nutritional recommendation" OR "nutrition recommendation" OR "nutritional therapy" OR "nutrition therapy" OR "enriched meal*" OR "ons" OR "fsmp" OR "sip feed" OR "sip nutrition" OR "oral nutrition")<br>Show less	62,032,663
#3	TITLE-ABS-KEY ("sarcopenia" OR "sarcopeni*" OR "myopeni*" OR "muscle loss" OR "muscle depletion" OR "muscle wasting" OR "muscle reduction" OR "reduced muscle" OR "depleted muscle" OR "muscle attenuation" OR "muscle alteration" OR "loss of muscle" OR "muscular atrophy" OR "muscular atrophy" OR "muscle atrophy" OR "muscle, skeletal" OR "skeletal muscle" OR "muscle mass" OR "psoas muscle" OR "fat free mass" OR "lean mass" OR "muscle index" OR "muscle size" OR "muscle thickness" OR "body composition" OR "body composition" OR "malnutrition" OR "malnutrition" OR "muscle strength" OR "muscle strength" OR "muscular strength" OR "muscle power" OR "muscle function" OR "hand strength" OR "hand strength" OR "handgrip strength" OR "grip strength" OR "muscle fatigue" OR "muscle fatigue" OR "muscle weakness" OR "muscle weakness" OR "muscular weakness")<br>Show less	622,527
#4	TITLE-ABS-KEY ("randomized controlled trial" OR "randomized" OR "placebo")	1,440,348
#5	#1 AND #2 AND #3 AND #4	899
